# Integrated multi‐omics profiling of high‐grade estrogen receptor‐positive, HER2‐negative breast cancer

**DOI:** 10.1002/1878-0261.13043

**Published:** 2021-07-29

**Authors:** Kang Wang, Lun Li, Sebastià Franch‐Expósito, Xin Le, Jun Tang, Qing Li, Qianxue Wu, Laia Bassaganyas, Jordi Camps, Xiang Zhang, Hongyuan Li, Theodoros Foukakis, Tingxiu Xiang, Jiong Wu, Guosheng Ren

**Affiliations:** ^1^ Department of Endocrine and Breast Surgery The First Affiliated hospital of Chongqing Medical University Chongqing Medical University China; ^2^ Key Laboratory of Molecular Oncology and Epigenetics The First Affiliated Hospital of Chongqing Medical University China; ^3^ Department of Oncology‐Pathology Karolinska Institutet Stockholm Sweden; ^4^ Department of Breast Surgery Fudan University Shanghai Cancer Center China; ^5^ Cancer Institute Fudan University Shanghai Cancer Center China; ^6^ Department of Oncology Shanghai Medical College Fudan University Shanghai China; ^7^ Gastrointestinal and Pancreatic Oncology Team Institut D'Investigacions Biomèdiques August Pi i Sunyer (IDIBAPS) Hospital Clínic de Barcelona Centro de Investigación Biomédica en Red de Enfermedades Hepáticas y Digestivas (CIBEREHD) Universitat de Barcelona Spain; ^8^ Liver Cancer Translational Research Group Liver Unit Institut D'Investigacions Biomèdiques August Pi i Sunyer (IDIBAPS) Hospital Clínic Centro de Investigación Biomédica en Red de Enfermedades Hepáticas y Digestivas (CIBEREHD) Universitat de Barcelona Spain; ^9^ Unitat de Biologia Cel·lular i Genètica Mèdica Departament de Biologia Cellular, Fisiologia i Immunologia Facultat de Medicina Universitat Autònoma de Barcelona Bellaterra Spain; ^10^ Breast Center Theme Cancer Karolinska University Hospital Stockholm Sweden

**Keywords:** endocrine‐resistant subgroup, ER^+^HER2^−^ breast cancer, histologic grade, hypomethylated loci, multi‐omics

## Abstract

Estrogen receptor‐positive and human epidermal growth factor receptor 2‐negative (ER^+^HER2^−^) breast cancer accounts for ~ 60–70% of all cases of invasive breast carcinoma. High‐grade ER^+^HER2^−^ tumors respond poorly to endocrine therapy. In this study, we systematically analyzed clinical and multi‐omics data to find potential strategies for personalized therapy of patients with high‐grade ER^+^HER2^−^ disease. Six different cohorts were analyzed, for which multi‐omics data were available. Grade III ER^+^HER2^−^ cases harbored higher proportions of large tumor size (> 5 cm), lymph node metastasis, chemotherapy use, and luminal B subtypes defined by PAM50, as compared with grade I/II tumors. DNA methylation (HM450) data and methylation‐specific PCR indicated that the cg18629132 locus in the *MKI67* promoter was hypermethylated in grade I/II cases and normal tissue, but hypomethylated in grade III cases or triple‐negative breast cancer, resulting in higher expression of *MKI67*. Mutations in *ESR1* and *TP53* were detected in post‐endocrine treatment metastatic samples at a higher rate than in treatment‐naive tumors in grade III cases. We identified 42 and 20 focal copy number events in nonmetastatic and metastatic high‐grade ER^+^HER2^−^ cases, respectively, with either MYC or MDM2 amplification representing an independent prognostic event in grade III cases. Transcriptional profiling within grade III tumors highlighted ER signaling downregulation and upregulation of immune‐related pathways in non‐luminal‐like tumors defined by PAM50. Recursive partitioning analysis was employed to construct a decision tree of an endocrine‐resistant subgroup (GATA3‐negative and AGR‐negative) of two genes that was validated by immunohistochemistry in a Chinese cohort. All together, these data suggest that grade III ER^+^HER2^−^ tumors have distinct clinical and molecular characteristics compared with low‐grade tumors, particularly in cases with non‐luminal‐like biology. Due to the dismal prognosis in this group, clinical trials are warranted to test the efficacy of potential novel therapies.

## Graphical abstract

High histologic grade is associated with significantly increased risk of breast cancer‐specific mortality among patients with ER^+^HER2^−^ tumors, but the cause for worse outcomes in this subset remains unknown. Here, we integrated the multi‐omics data to reveal important biomarkers, therapeutic targets, and precision medicine strategy for grade III ER^+^HER2^−^ breast cancer patients.

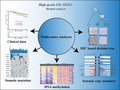

**Abbreviations**
CI: confidence intervals
ER: estrogen receptor
FUSCC: Fudan University Shanghai Cancer Center
HER2: human epidermal growth factor receptor 2
HRs: hazard ratios
IHC: immunohistochemistry
METABRIC: Molecular Taxonomy of Breast Cancer International Consortium
MSKCC: Memorial Sloan Kettering Cancer Center
MSP: methylation‐specific PCR
PR: progesterone receptor
RPA: recursive partitioning analysis
TCGA: The Cancer Genome Atlas
TNBC: triple‐negative breast cancer
WCCCG: Western China Clinical Cooperation Group

## 1 Introduction

Breast cancer is classified into different molecular subtypes based on estrogen receptor (ER), progesterone receptor (PR), and human epidermal growth factor receptor 2 (HER2) status, which dictate distinct therapeutic choices and clinical outcomes [1]. Histologic grade, representing degree of tumor cell differentiation (tubule formation, nuclear pleomorphism, and mitotic count), is one of the best‐established prognostic factors in breast cancer, but has a differential effect on survival for each molecular subtype [2]. Previous studies have demonstrated that the maximum benefit of histologic grade assessment would be in the subgroup of patients with early ER‐positive/HER2‐negative (ER^+^HER2^−^) tumors [3, 4, 5, 6, 7, 8], which contributes to 60–70% of all breast cancer [9]. A series of studies highlighted that histologic grade is an independent prognostic factor for patients with ER^+^HER2^−^ tumors in gene signature‐based models [2, 10, 11]. Notably, high histologic grade might be associated with resistance to endocrine therapy in node‐negative ER^+^HER2^−^ subgroup, when only receiving adjuvant endocrine therapy, with 7% rate of 10‐year risk of relapse for grade I, 14% for grade II, and 31% for grade III tumors [5].

Given substantial inter‐ and intralaboratory variation in histologic grading [12], numerous studies investigating the molecular basis of morphological phenotypes in breast cancer and its integration with molecular data have identified room for improvement in terms of breast cancer classification, therapy response prediction, and clinical management [13, 14, 15, 16]. Several genetic abnormalities are found to be statistically associated with higher histologic grade in invasive breast cancer, including three most prevalent cancer driver events, *TP53* and *PIK3CA* mutations and *MYC* amplification [16, 17, 18, 19, 20]. Transcriptomic profiles at gene and isoform level can be used to stratify grade II tumors into two distinct groups with different prognostic outcomes, which has the potential to reduce both under‐ and overtreatment of breast cancer patients [14]. Nevertheless, previous omics‐based investigations have not considered the role of molecular subtype when studying histologic grade in breast cancer [3, 21], especially for ER^+^HER2^−^ subtype.

High histologic grade is associated with significantly increased risk of breast cancer‐specific mortality among patients with ER^+^HER2^−^ tumors, but the cause for worse outcomes in this subset remains unknown. Therefore, a comprehensive assessment based on multi‐omics data is needed in order to provide evidence of individualized decision‐making for those high‐grade ER^+^HER2^−^ cases. In this study, we analyzed six different breast cancer cohorts aiming to characterize clinicopathological features, epigenetic regulation factors, genomic alterations, and develop gene panel to identify its intrinsic molecular subtypes, which may serve as novel biomarkers or therapeutic targets for grade III ER^+^HER2^−^ breast cancer patients’ treatments.

## 2 Methods

### 2.1 Patients and samples

This study included six cohorts comprising clinicopathological, multi‐omics, and follow‐up data. Patients’ selection included ER^+^HER2^−^ status with known nuclear grade. Additionally, the study also included triple‐negative breast cancer (TNBC) cases, as a comparison because of their HER2‐negative status and worse survival in breast cancer. The first longitudinal cohort study used the April 2019 release of the Surveillance, Epidemiology, and End Results (SEER) database of the National Cancer Institute (NCI). This database included 18 population‐based cancer registries covering 34.6% of the US population [22]. Of 150 060 enrolled HER2‐negative patients, 25 629 (17.1%) were grade III ER^+^HER2^−^. The second and third cohorts were the Molecular Taxonomy of Breast Cancer International Consortium (METABRIC) and The Cancer Genome Atlas (TCGA) including 404 and 88 grade III ER^+^HER^−^ cases, respectively, with RNA‐seq, somatic mutation, copy number alterations (CNAs), and clinical data. The pathway or signature scores were obtained for breast cancer samples within TCGA from the supplementary table of Perou and colleagues, which can be accessed by the link: https://www.ncbi.nlm.nih.gov/pmc/articles/PMC4603750/bin/NIHMS724218‐supplement‐2.xlsx. The fourth study was from Memorial Sloan Kettering Cancer Center (MSKCC) [23], containing 272 nonmetastatic and 39 metastatic grade III ER^+^HER^‐^ patients with somatic mutation and CNA data. The fifth and sixth cohorts are from China including 546 and 348 grade III ER^+^HER2^−^ patients obtained from Western China Clinical Cooperation Group (WCCCG) and Fudan University Shanghai Cancer Center (FUSCC), respectively.

Treatment‐naïve breast cancer tissues and adjacent normal breast tissues were obtained from patients who had undergone surgery at the Department of Endocrine and Breast Surgery, the First Affiliated Hospital of Chongqing Medical University. All samples were stored at −80 °C until evaluation by pathologists. All tumor sample tissues were macrodissected with 50–70% of tumor cells. All participants provided written consent before enrollment, and the research was approved (ref #2020‐311) by Institutional Ethics Committees of the First Affiliated Hospital of Chongqing Medical University. All procedures performed in studies involving human participants were in accordance with the ethical standards of the institutional and/or national research committee and with the 1964 Helsinki Declaration and its later amendments or comparable ethical standards.

### 2.2 Multi‐omics analyses

#### 2.2.1 Somatic copy number alterations

We applied GISTIC2.0 (genomic identification of significant targets in cancer) to identify regions of copy number alteration, which is a statistical method that calculates a score that is based on both amplitude and frequency of copy number changes at each position in the genome, using permutation testing to determine significance [24]. Significant focal regions of amplification and deletion were identified by applying GISTIC with following parameters (‐ta 0.2 ‐td 0.2 ‐genegistic 1 ‐smallmem 1 ‐broad 1 ‐conf 0.95 ‐rx 0 –brlen 0.7 ‐cap 2.5 –armpeel 1). The CNA events were defined according to the discrete copy number calls provided by GISTIC 2.0: −2 = homozygous deletion; −1 = hemizygous deletion; 0 = neutral; 1 = gain; and 2 = amplification. In addition, CNApp (https://github.com/elifesciences‐pub‐lications/CNApp), developed by our group, was used to compute CNA scores based on the number, length, and amplitude of broad and focal genomic alterations, to assess differentially altered genomic regions, and to perform machine learning‐based predictions to classify high and low/intermediate grade ER^+^HER2^−^ tumors. First, CNApp applies a resegmentation approach to adjust for amplitude divergence due to technical variability and correct for estimated tumor purity. Resegmented data are then used to calculate the broad (BCS), focal (FCS), and global (GCS) CAN scores, which provide three different quantifications of CNA levels for each sample. To compute these scores, CNApp classifies and weights CNAs based on their length and amplitude. For each sample, BCS is computed by considering broad (chromosome and arm‐level) segment weights according to the amplitude value. Likewise, calculation of FCS takes into account weighted focal CNAs corrected by the amplitude and length of the segment. Finally, GCS is computed by considering the sum of normalized BCS and FCS, providing an overall assessment of the CNA burden. We downloaded segmented CAN data of TCGA and MSKCC cohorts form cbioportal (http://www.cbioportal.org). Mapping information for CNA, Refgene and cytoband locations are based on the hg19 build of the human genome sequence from the University of California, Santa Cruz (http://genome.ucsc.edu).

#### 2.2.2 Somatic mutation

Somatic mutation data of the TCGA and METABRIC cohort were acquired from the ‘data_mutations_extended’ file downloaded from cBioPortal (http://www.cbioportal.org). We employed chi‐square test or Fisher's exact test to compare the mutation rates between grade I/II ER^+^HER2^−^, grade III ER^+^HER2^−^, and TNBC cases. Furthermore, mutational signatures were, respectively, estimated in three groups, and extracted signatures can also be compared to those validated signatures (https://cancer.sanger.ac.uk/cosmic/signatures). APOBEC‐induced mutations are more frequent in solid tumors and are mainly associated with C>T transition events occurring in TCW motif. APOBEC enrichment scores in the above command are estimated using the method described by Roberts *et al*. [25]. The further analyses and visualizations were conducted according to the workflow of Bioconductor package ‘maftools’ (https://bioconductor.org/packages/release/bioc/vignettes/maftools/inst/doc/maftools.html).

### 2.3 DNA methylation (HM450)

We employed the strategies of ELMER v.2 [26] to process the DNA methylation (HM450) data of TCGA cohort downloaded by Bioconductor package ‘TCGAbiolinks’ from Genomic Data Commons (GDC) [27], which provides a systematic approach that reconstructs altered gene regulatory networks by combining enhancer methylation and gene expression data derived from the same sample set using MultiAssayExperiment (MAE) data structure. The methylation level of CpGs was represented as β values (β = Intensity of the methylated allele (M)/[Intensity of the unmethylated allele (U) + Intensity of the methylated allele (M) + 100], ranging from 0 to 1). ELMER first identifies differentially methylated CpGs (DMCs) occurring at promoter probes within two comparisons (grade III ER^+^HER2^−^ vs normal tissue; grade III ER^+^HER2^−^ vs grade I/II ER^+^HER2^−^) and then searches for up/downstream gene targets for each DMC. For each probe‐gene pair tested, the raw *P*‐value Pr was corrected for multiple hypothesis using a permutation approach.

### 2.4 GSVA/GSEA

Gene set enrichment analyses (GSEAs) were performed using the gsea software (v 4.0.3) [28] and the Molecular Signature Database (v 6.1; http://www.broad.mit.edu/gsea/) using the GSEA preranked function. One thousand total permutations were used. The ‘‘gsva’’ function in the r package ‘‘GSVA’’ [29]was used to calculate the pathway scores.

### 2.5 Development of IHC classifier

We constructed an immunohistochemistry (IHC) classifier to identify non‐luminal‐like cases in grade III ER^+^HER2^−^ patients. Firstly, differential gene expression (DGE) analyses were conducted between grade non‐luminal‐like and luminal‐like III ER^+^HER2^−^ cases, setting parameters as |Fold change| > 2 and adjusted *P* < 0.05. Among 641 DGEs, 386 genes upregulated and 255 genes downregulated. To ascertain it tested by IHC in clinical practice, we only kept genes that had evidence of protein level in The Human Protein Atlas (TCPA; http://www.proteinatlas.org/), where we excluded 182 genes. Then, we further selected 184 genes that had positive correlations (correlation coefficient < 0.5, *P* < 0.05) with their proteins based on CPTAC database (https://proteomics.cancer.gov/programs/cptac). At last, a nonparametric classification recursive partitioning analysis (RPA) model was constructed based on 184 DGEs. The expressions of GATA3 and AGR3 were identified as joint determinants for non‐luminal‐like cases. Also, the prediction ability and clinical significance of this IHC classifier was validated using METABRIC cohort.

### 2.6 Methylation‐specific PCR

To evaluate *MKI67* methylation status, methylation‐specific PCR (MSP) was performed by using AmpliTaq‐Gold DNA Polymerase (Applied Biosystems) [30]. Vector was used as a loading control. MSP products were separated on 2% agarose gels (MBI Fermentas, Vilnius, Lithuania) and photographed on a gel imaging system (Bio‐RAD Gel Doc XR^+^, CA, USA). The methylation‐specific primers were ascertained based on location of cg18629132 and shown in Table S1.

### 2.7 Immunohistochemistry

To evaluate the expression levels of *GATA3* and *AGR3* in tumor tissues of grade III ER^+^HER2^−^ patients, IHC was performed using anti‐GATA3 (sc‐269; Santa Cruz) and anti‐AGR3 (sc‐390940; Santa Cruz). Tissue sections were fixed with 4% formaldehyde and embedded with paraffin. The expression levels (i.e., positive or negative) were assessed by mean density using image pro plus software.

### 2.8 Statistical analysis

To present the demographic, clinicopathological, and follow‐up characteristics of the study cases, mean and standard deviation (SD) values for continuous variables that are non‐normally distributed as indicated by Shapiro–Wilk normality test (all *P* < 0.05) and frequencies (percentages) for categorical variables were calculated. Kruskal–Wallis tests were conducted for non‐normally distributed continuous variables, and two‐sided Fisher exact tests or chi‐square tests were used to analyze contingency tables.

For outcomes in this study, patients were followed from diagnosis of primary invasive breast cancer until cancer relapse, death, loss to follow‐up, or the end of follow‐up. Disease‐free survival (DFS) was defined as days from breast cancer diagnosis until confirmation of cancer recurrence or death. Overall survival (OS) was defined as an interval from breast cancer diagnosis to death from any cause. Breast cancer‐specific survival (BCSS) was defined as the time from diagnosis to death from breast cancer. Cox proportional risk modeling was fitted to estimate crude and multivariable‐adjusted hazard ratios (HRs) and 95% confidence intervals (CI). To minimize the potential impacts of competing risk bias, competing risk regression models were employed to estimate subdistribution HR and 95% CI of BCSS, with nonbreast cancer causes of death as competing risk events [31]. The Kaplan–Meier method was used to estimate plotted survival probabilities, whose *P* values for differences between survival curves were calculated using the log‐rank test.

All statistical analyses were conducted using r‐3.6.1 (https://www.r‐project.org/), and the *P* values were two‐sided. *P* values of < 0.05 were considered statistically significant. For multiple testing adjustment, a false discovery rate (FDR) was calculated.

## 3 Results

### 3.1 Clinicopathological characteristics and survival outcomes of grade III ER^+^HER2^−^ tumors

As shown in Tables S2–S4, compared with cases with grade I/II ER^+^HER2^−^ tumors, grade III ER^+^HER2^−^ patients had younger age at diagnosis, more intrinsic luminal B‐like subtypes, invasive ductal carcinomas, larger tumors, greater risk of lymph node metastasis, and a higher chance of receiving chemotherapy.

**Fig. 1:**
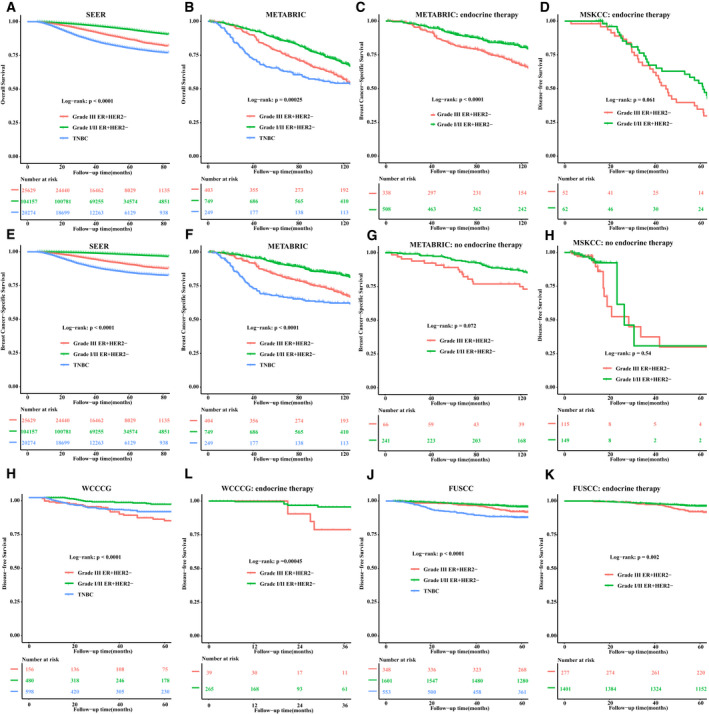
Survival analysis between histologic grade I/II ER^+^HER2^−^, III ER^+^ HER2^−^, and TNBC breast cancer. Kaplan–Meier curves of OS from the SEER (A) and the METABRIC cohort (B); BCSS within grade III ER^+^HER2^−^ patients who received endocrine therapy group from the METABRIC (C) and MSKCC cohort (D); BCSS from the SEER (E) and METABRIC cohort (F); BCSS within grade III ER^+^HER2^−^ patients who did not receive endocrine therapy group from the METABRIC (G) and MSKCC cohort (H); DFS from WCCCG (I) and FUSCC (K); DFS within grade III ER^+^HER2^−^ cases who received more than 1‐year endocrine therapy from WCCCG (J) and FUSCC (L).

After the full adjustment for confounders, patients with grade III ER^+^HER2^−^ tumors had worse intermediate survival outcomes compared with grade I/II ER^+^HER2^−^ cases or TNBC, including DFS [multivariate HR (95% CI) ER^+^HER2^−^ grade III vs grade I/II, MSKCC, 2.08 (1.22–3.56), *P* < 0.001; WCCCG, 2.52 (1.32–4.81), *P* < 0.001; FUSSC, 1.56 (1.00–2.44), *P* = .04; Fig. 1; Table S5], OS [multivariate HR (95% CI) ER^+^HER2^−^ grade III vs grade I/II, SEER, 1.95 (1.85–2.04), *P* < 0.001; METABRIC, 1.20 (1.00–1.45), *P* = 0.04; Fig. 1; Table S6], and BCSS [multivariate HR (95% CI) ER^+^HER2^−^ grade III vs grade I/II, SEER, 3.06 (2.85–3.27), *P* < 0.001; METABRIC, 1.79 (1.44–2.22), *P* < 0.001; Fig. 1; Table S6].

To systematically assess interaction effect of molecular subtype and histologic grade on BCSS, we conducted survival analyses for all nonmetastatic, female breast cancer patients diagnosed between 2010 and 2014 in SEER cohort. When observing the association between histologic grade and BCSS, this differed between the subgroups stratified by IHC‐defined molecular subtypes (i.e., ER^+^HER2^−^, ER^+^HER2^+^, ER^−^PR^−^HER2^+^, and TNBC; Table S7, *P*_interaction_ < 0.001). Higher histologic grade was strongly associated with an increased risk of mortality in subjects with ER^+^HER2^−^ tumors (multivariate HR grade III vs grade I, 4.31; 95% CI, 3.85–4.82; *P*_trend_, < 0.01; Fig. 1E; Table S7), but moderate (ER^+^HER2^+^ and TNBC) and no associations (ER^−^PR^−^HER2^+^) were found in other phenotypes.

In the ER^+^HER2^−^ subsets receiving endocrine therapy, grade III cases were associated with worse DFS compared with their grade I/II counterparts (Fig. 1C,D,H,K), but there was no difference in DFS between grade III and I/II cases who did not receive endocrine therapy (Fig. 1G,H).

### 3.2 Overview of multi‐omics profiling data

**Fig. 2:**
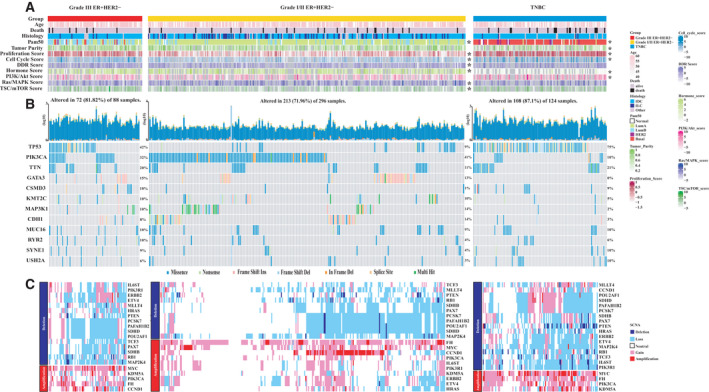
The genomic landscape of HER2‐negative breast cancers from the TCGA. (A) HER2‐negative samples are classified into three groups according to the IHC‐based ER status and histologic grade. Clinical and molecular features are annotated below. (B) Waterfall plot showing the somatic mutations that affected the most frequently altered genes (rows). (C) CNAs affecting cancer genes (significant GISTIC peaks with residual *q* < 1 × 10^−4^) as defined by The Sanger Institute: Cancer Gene Census (https://cancer.sanger.ac.uk/census). *T test or Pearson's chi‐square test indicating statistically difference with histologic grade III ER^+^HER2^−^ patients.

Patients with grade III ER^+^HER2^−^ presented higher oncogenic pathway score proliferation, cell cycle, DNA damage response score, and lower hormone score than those with grade I/II ER^+^HER2^−^ cases (all *P* < 0.05, see Fig. 2A and Table S2). Somatic alterations, including gene‐level mutations and CNAs, were compared between three groups (Fig. 2B,C and Figs S1 and S2). Notably, known cancer driver events such as mutated *TP53* (42.0% vs 8.8%) and *RB1* loss (60.9% vs 35.8%) were enriched in grade III cases compared to grade I/II cases. Mutation signatures represent characteristic mutation patterns of DNA damage as a result of interplay between exogenous or endogenous mutagenic agents and DNA repair system [32]. In order to identify mutagenic agents specifically present in grade III ER^+^HER2^−^ patients, we conducted signature extraction and compared them to known signatures from COSMIC database [33]. The mutational signatures of grade III ER^+^HER2^−^ patients were found to be similar with Single Base Substitution (https://cancer.sanger.ac.uk/signatures/sbs/) Fig. S1–S3, where signature 3 was associated with defective DNA double‐strand break‐repair system by homologous recombination.

### 3.3 Genome‐wide DNA methylation profiling of histologic grade III ER^+^HER2^−^ patients

**Fig. 3:**
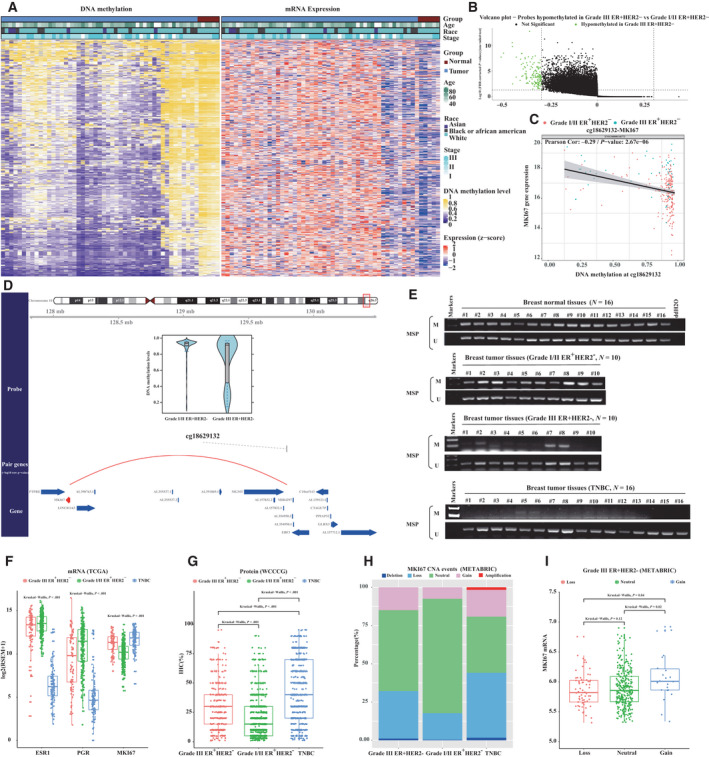
Genome‐wide DNA methylation profiling of histologic grade III ER^+^HER2^−^ patients. (A) Overall summary of differential DNA methylation levels between histologic grade III ER^+^HER2^−^ tumors with normal samples, and their corresponding gene expression levels. (B) Volcano plot showing the DNA methylation probes hypomethylated within histologic grade III ER^+^HER2^−^ patients compared with I/II ER^+^HER2^−^ cases. (C) Correlation between mRNA MKI67 expression and DNA methylation levels at cg18629132 by Pearson's correlation test. (D) Schematic plot showing relationship between the probe‐gene pairs inferred. (E) cg18629132 methylation within primary normal breast tissues (*n* = 16), histologic grade III ER^+^HER2^−^ tumor tissues (*n* = 10), histologic grade I/II ER^+^HER2^−^ tumor tissues (*n* = 10), and TNBC tissues (*n* = 16), measured by MSP. M, methylated and U, unmethylated, where the experiments were replicated twice. (F) Log_2_‐transformed mRNA expression levels (RSEM) of ESR1, PR, and MKI67. *P*‐value was calculated by the Mann–Whitney test. (G) Protein levels (IHC, %) within histologic grade III ER^+^HER2^−^ tumors, I/II ER^+^HER2^−^ tumors, and TNBC from WCCCG. *P*‐value was calculated by the Mann–Whitney test. (H) Distribution of the copy deletion, loss, neutral, gain, and amplification group within histologic grade III ER^+^HER2^−^ tumors, I/II ER^+^HER2^−^ tumors, and TNBC. (I) Log_2_‐transformed mRNA expression levels (RSEM) of MKI67 between the copy loss, neutral, and gain group within histologic grade III ER^+^HER2^−^ tumors.

To elucidate epigenetic alterations and their roles among grade III ER^+^HER2^−^ patients, we attempted to identify significantly hyper/hypomethylated genes by integrating results from DNA methylation assay produced by Illumina Infinium HumanMethylation450 BeadChip platform (HM450) and RNA‐seq data from TCGA consortium. Subsequent comparisons were assessed: (a) grade III vs grade I/II ER^+^HER2^−^ tumors and (b) grade III ER^+^HER2^−^ tumors vs normal tissues. We first identified DMCs occurring at promoter probes and searched for downstream gene targets for each DMC (Fig. S4). DNA methylation data from grade III (*n* = 54) and I/II ER^+^HER2^−^ (*n* = 202) tumors were used in the differential methylation analysis (|β value difference| > 0.3 and FDR < 0.05). Compared with grade I/II cases, patients with grade III ER^+^HER2^−^ cases harbored 17 hypermethylated and 135 hypomethylated DMCs, corresponding to 5 hypermethylated and 14 hypomethylated genes (Table S8 and Fig. S5). When comparing normal tissues (*n* = 14), 11 085 hypermethylated and 14 829 hypomethylated DMCs were, respectively, associated with 678 downregulated and 3726 upregulated mRNA expression of genes in grade III ER^+^HER2^−^ cases (Fig. 3A and Fig. S5). The only overlapped CpG‐gene pair, *cg18629132‐MKI67* (grade III vs grade I/II ER^+^HER2^−^, mean β value difference = −0.41, FDR < 0.001; mRNA logFC = 1.15; FDR < 0.001; Fig. 3B,C and Fig. S6; grade I/II ER^+^HER2^−^ vs normal tissues, mean β value difference = −0.49, FDR < .001; mRNA logFC = 3.65; FDR < 0.001), between assessed comparisons was revealed, when only considering CpG‐gene pairs with statistically significant FDR values. This result suggested that grade III ER^+^HER2^−^ cases may lose DNA methylation in the process of differentiation, contributing to proliferation of tumors by epigenetically upregulating MKI67 expression.

To validate these prior results, we performed the MSP analysis on specimens from the First Affiliated Hospital of Chongqing Medical University, where we had access to 10 grade I/II ER^+^HER2^−^, 10 grade III ER^+^HER2^−^, 15 TNBC tumors, and 15 normal breast tissues. The methylation in *MKI67* promoter was detected in all grade I/II ER^+^HER2^−^ tumors (10/10) and normal breast tissue samples (15/15), but in only 3 out of 10 (30%) grade III ER^+^HER2^−^ tumors and 0 out of 15 TNBC tissues (Fig. 3E).

We further confirmed that grade III ER^+^HER2^−^ tumors were associated with higher mRNA and protein expression level of *MKI67* than those with grade I/II tumors in the TCGA and WCCCG cohorts, respectively (Fig. 3F,G). We also found more copy number gain events overlapping the *MKI67* genomic region in patients with grade III ER^+^HER2^−^ than those with grade I/II tumors, potentially contributing to high mRNA expression of *MKI67* (Fig. 3H,I). Interestingly, the mRNA *MKI67* expression level interacted with nuclear grade on survival, suggesting ER^+^HER2^−^ patient with grade III and high *MKI67* expression harbored worst BCSS (Fig. S7).

### 3.4 CNA profiling of histologic grade III ER^+^HER2^−^ tumors

**Fig. 4:**
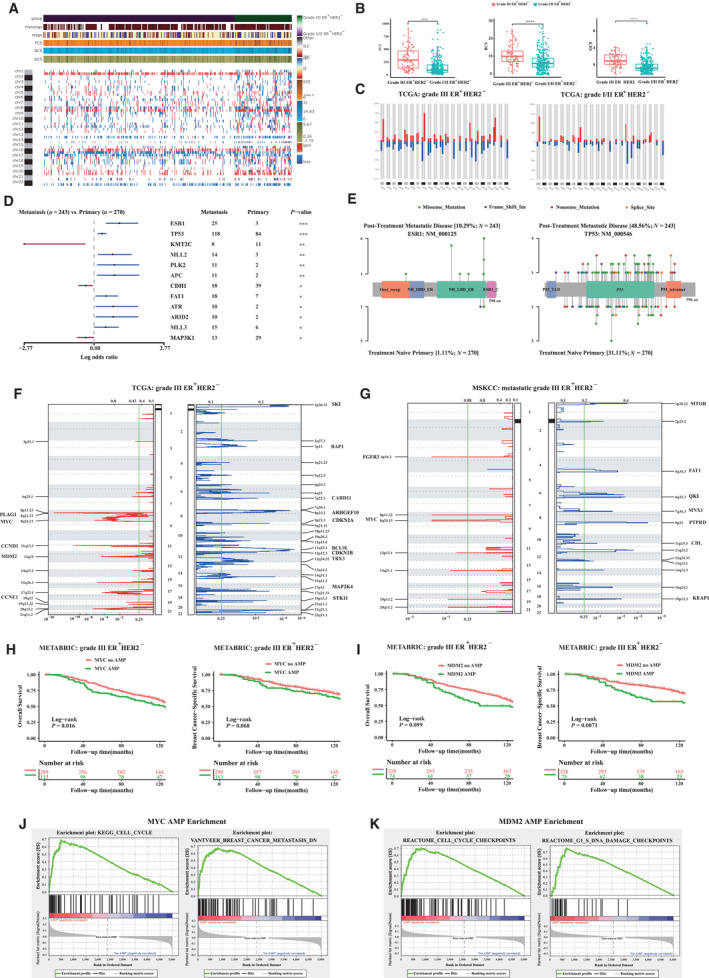
Elucidating therapeutic molecular targets for histologic grade III ER^+^HER2^−^ breast cancer patients. (A) Genome‐wide profiling by chromosome arms distributed according to the histologic grade. (B) FCS, BCS, and GCS distribution by the histologic grade. (C) CNApp frequencies for chromosome arm regions using default cutoffs, corresponding to 2.3/1.7 copies for gains and losses, respectively. (D) Forest plot showing the tumor evolution under endocrine therapy (Pearson’s chi‐square or Fisher exact test; ***P < 0.001, **P < 0.01, and *P < 0.05), where somatic mutations within treatment‐naïve primary disease were compared with that of metastatic disease from MSKCC cohort. (E) Two top differential mutated genes (ESR1 and TP53) between treatment‐naïve primary disease and metastatic disease discovered in MSKCC cohort. Mutations were labeled in a diagram of the gene coding region, and the heights of the ‘‘lollipop’’ sticks indicate the number of the indicated mutation. (F) GISTIC plots. Regions of gain and loss delineated by GISTIC analysis of grade III ER^+^HER2^−^ breast cancer samples from TCGA cohort. Significance is reported as false discovery rate‐corrected *q*‐value. Known tumor suppressor genes and proto‐oncogenes defined as found in COSMIC; if there is more than one known proto‐oncogene in the region, only one is listed (priority for listing is, in order: known breast mutation; other known mutation (by COSMIC frequency). (G) GISTIC plots. Regions of gain and loss delineated by GISTIC analysis of metastatic grade III ER^+^HER2^−^ breast cancer cases from MSKCC cohort. (H) Kaplan–Meier curves of OS and BCSS between grade III ER^+^HER2^−^ breast cancer patients with MYC amplification and non‐MYC amplification. (I) Kaplan–Meier curves of OS and BCSS between grade III ER^+^HER2^−^ breast cancer patients with MDM2 amplification and non‐MDM2 amplification. (J) Enriched pathways related to MYC amplification and (K) related to MDM2 amplification within grade III ER^+^HER2^−^ breast cancer from the TCGA cohort by gene set enrichment analysis (GSEA).

The CNApp tool [34] (https://github.com/elifesciences‐pub‐lications/CNApp) previously developed by Sebastià *et al*. was used to compute CNA scores based on the number, length, and amplitude of broad and focal genomic alterations and to assess differentially altered genomic region. Accordingly, the broad (BCS), focal (FCS), and global (GCS) CNA scores were, respectively, calculated, providing three different quantifications of CNA levels for each sample (see details in Appendix S1). Histologic grade III ER^+^HER2^−^ tumors harbored higher FCS (median value of 296 vs 94), BCS (median value of 40.5 vs 10), and GCS (median value of 0.95 vs −0.72) than those with low‐grade ER^+^HER2^−^ tumors (Fig. 4A,B). Considering that cancer type‐specific patterns of genomic gains and losses determined the tissue‐of‐origin [35], we conducted subsequent analysis aimed at generating genome‐wide patterns for each group based on chromosome‐arm genomic windows and the overall corresponding frequencies. Chromosome arms altered in more than 25% across low/intermediate and high histologic grade ER^+^HER2^−^ tumors were 1q, 8q, 16p, and 20q for copy number gains, and 8p, 13q, 16q, 17p, and 22q for copy number losses. Moreover, the top five distinctive chromosome arms affected by CNAs between histologic grade III and I/II tumors included chromosome arms 8q (75.9% vs 39.2%), 20p (38.0% vs 18.2%) and 20q (62.1% vs 26.1%), and 9p (36.8% vs 15.1%) and 14q (25.3% vs 6.5%) for copy number gains and for copy number losses.

### 3.5 Identification of candidate driver events in grade III ER^+^HER2^−^ tumors

We sought to identify candidate driver events for patients with grade III ER^+^HER2^−^tumors based on treatment‐naive primary (*n* = 270) and post‐treatment metastatic diseases (*n* = 243) from MSKCC cohort. Mutation enrichment in genes *ESR1* (10.3% vs 1.1%) and *TP53* (48.6% vs 31.1%) was detected in metastatic samples with aromatase inhibitor treatment compared to treatment‐naive tumors (Fig. 4D; Tables S9–S12). The majority of the *TP53* alterations in patients with metastatic disease were missense mutations located in the DNA binding domain, while some were also present in the tetramerization domain (Fig. 4E).

To identify regions of CNA, we applied GISTIC (genomic identification of significant targets in cancer) [36], a statistical method that calculates a score that is based on both the amplitude and frequency of copy number changes at each position in the genome. GISTIC identified 16 oncogenic focal events in grade III ER^+^HER2^−^ cases from TCGA (Fig. 4F and Fig. S8; Table S13) and 10 focal events in metastatic grade III ER^+^HER2^−^ from MSKCC (Fig. 4G and Fig. S8; Table S14). The overlap among two cohorts is only limited to the amplification of *MYC* on 8q24.13/8q24.21, which was found in 46.6% and 42.3% of samples from TCGA and MSKCC cohorts, respectively. The enrichment of specific focal events in grade III ER^+^HER2^−^ patients was also observed in the METABRIC cohort, where amplification events of *CCND1* (26.8% vs 13.3%), *MYC* (28% vs 12.4%), and *MDM2* (18.6% vs 7.4%) were present when compared with grade I/II cases (Tables S15 and S16).

In the METABRIC cohort, multivariate Cox proportional hazards regression adjusted for age at diagnosis, tumor stage, radiotherapy, endocrine therapy, chemotherapy, and surgery indicated that *MDM2* amplification was an independently prognostic factor on OS (HR AMP vs no AMP, 1.40, 95% CI, 1.01–1.96, *P* = 0.045; Fig. 4H and Table S17) and BCSS (HR AMP vs no AMP, 1.72, 95% CI, 1.17–2.53, *P* = 0.006; Fig. 4F and Table S17). Similarly, there was a trend toward shorter OS in grade III ER^+^HER2^−^ patients with *MYC* amplification compared with those without (HR AMP vs no AMP, 1.26, 95% CI, 0.95–1.68, *P* = 0.11; Fig. 4I). When assessing enrichment pathways in the *MDM2/MYC* amplification group by GSEA within grade III ER^+^HER2^−^ breast cancer, we identified that grade III ER^+^HER2^−^ patients with *MDM2/MYC* amplification were enriched in cell cycle *KEGG* pathway (Fig. 4J,K, Tables S18 and S19). Specifically, amplification events of *MYC* were associated with higher expression of cell cycle‐related genes (i.e., *CCNE2*, *MKI67*) compared to patients without *MYC* amplification. No association between *MYC* copy number status and *TP53* mutation status within grade III ER^+^HER2^−^ tumors was observed (Figs S9 and S10), indicating that *MYC* amplification correlated with *TP53*‐independent cell cycle progression. Additionally, significantly enriched DNA damage checkpoint gene sets were related to *MDM2* amplification (Fig. 4K; Table S19).

### 3.6 Therapeutic response of grade III ER^+^HER2^−^ breast cancers differs according to the intrinsic subtypes

**Fig. 5:**
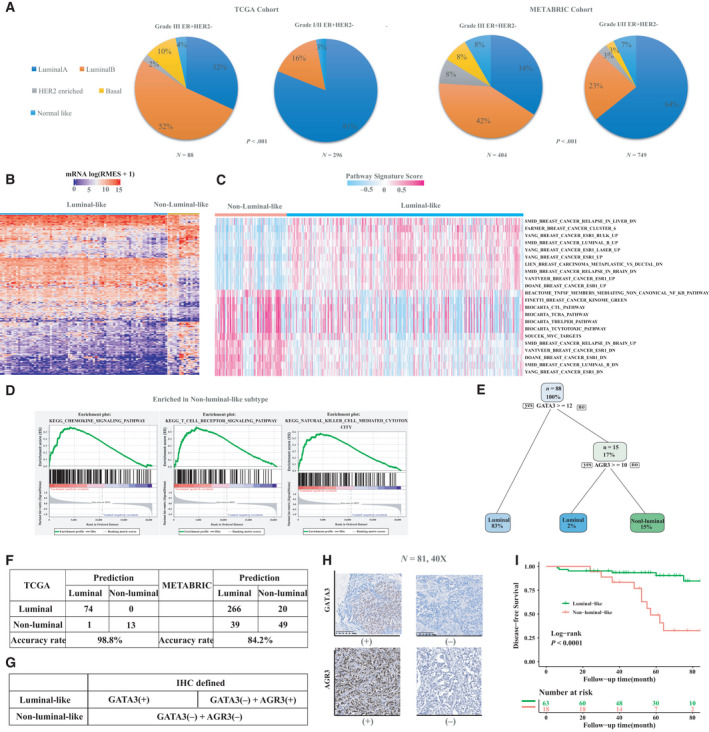
An IHC‐based model to identify PAM50 nonluminal intrinsic disease in histologic grade III ER^+^HER2^−^ breast cancer. (A) Intrinsic subtypes distribution between high and low/intermediate grade ER^+^HER2^−^ tumors. *P* values were calculated based on Pearson's chi‐squared tests. (B) Analyzing differential gene expression between non‐luminal‐like and luminal‐like grade III ER^+^HER2^−^ cases. (C) Differential pathways in nonluminal grade III ER^+^HER2^−^ tumors in C2 sets (curated sets) by GSVA (FDR < 0.05, |logFC|> 0.2) compared with counterparts with luminal‐like tumors. (D) Enriched pathways related to chemokine and T‐cell receptor signaling pathways, and natural killer cell mediated cytotoxicity within non‐luminal‐like grade III ER^+^HER2^−^ tumors. (E) RPA‐generated non‐luminal‐like stratification of patients with grade III ER^+^HER2^−^ tumors from TCGA. (F) Confusion Matrix of 2‐gene classifier in TCGA database and METABRIC database. (G) Criteria of identification of non‐luminal‐like subtype in grade III ER^+^HER2^−^ cases. (H) Representative images of GATA3 and AGR3 immunohistochemical staining (*N* = 81, 40×), where the experiments were replicated twice. (I) Comparison of disease‐free survival between the non‐luminal‐like and the luminal‐like subgroup.

Considering intrinsic molecular profiling (PAM50) provides additional prognostic information for early‐stage ER^+^HER2^−^ breast cancers [37, 38], we compared intrinsic subtypes distribution between grade I/II and III ER^+^HER2^−^ tumors. There are more luminal B‐like subtype and non‐luminal‐like subtypes (i.e., normal‐like, HER2 enriched and basal‐like) among grade III ER^+^HER2^−^ breast cancer patients than those with grade I/II tumors (Fig. 5A), indicating that high‐grade tumors are heterogeneous. In addition, given that intrinsic luminal A and B subtypes predict 10‐year outcome [38], we grouped ER^+^HER2^−^ breast cancer into intrinsic luminal‐like and nonluminal cases.

There were 74 (84.1%) luminal cases and 14 (15.9%) nonluminal cases in patients with grade III ER^+^HER2^−^ from TCGA cohort. Similarly, a quarter of grade III ER^+^HER2^−^ patients (24.0%) from METABRIC were enriched in nonluminal subtypes. To identify molecular differences between the nonluminal and luminal subtype, we performed differential gene expression analysis between the two groups of tumors, identifying a total of 641 differentially expressed genes (FDR < 0.05, |logFC| > 2; Fig. 5B and Table S20), with 255 of these upregulated in nonluminal and 386 upregulated in luminal subtype. We then computed GSVA scores of 6475 known pathways and performed similar differential comparisons between non‐luminal‐like and luminal‐like tumors from III ER^+^HER2^−^ patients. We found that 147 statistically differential pathways were upregulated in non‐luminal‐like tumors and 25 were upregulated in luminal‐like tumors (FDR < 0.05, |logFC| > 0.2; Fig. 5C; Tables S21 and S22). In brief, 10 out of the 22 top differential pathways were associated with ER^+^ factors, further supporting the resistance mechanism to endocrine therapy that ER signaling is a rarer driver in non‐luminal‐like compared to luminal group within grade III ER^+^HER2^−^ patients. T‐cell markers, epithelial‐to‐mesenchymal transition, TNF signaling via NF‐kB pathway, cytokine signaling genes as well as immune‐related pathways such as PD‐1, CD8, IL‐2, and IL‐12 signaling pathways, and immune cell type signatures such as cytotoxic cells and NK cells were upregulated in the nonluminal group (Fig. 5C,D).

To shed light on non‐luminal‐like predictive panel in the clinical practice, we conducted recursive partitioning analyses using optimized binary partition algorithm based on 641 differentially expressed genes in the TCGA cohort. Detailed genes' selection strategy is shown in Fig. S11, where genes with evidence at protein level and satisfying correlation between mRNA and protein expression were considered as candidates (Fig. S12). We developed a two‐gene panel (*GATA3* and *AGR3*) to identify non‐luminal‐like cases within grade III ER^+^HER2^−^ patients, where both *GATA3* and *AGR3* tended to associate with the luminal‐like subtype (Fig. 5E). The confusion matrix of two‐gene classifier in TCGA database and METABRIC database indicated that our panel harbored 98.8% and 84.2% accuracy for the training (TCGA) and validation (METABRIC) sets, respectively (Fig. 5F and Fig. S13). Additional cross‐validation procedures were processed within METABRIC cohort, and we observed the survival difference between luminal and nonluminal cases identified by our two‐gene panel in the endocrine therapy subgroups. Specifically, when receiving endocrine therapy, nonluminal cases had inferior BCSS compared to luminal‐like patients, but the survival difference disappeared if they did not receive endocrine therapy (Fig. S14).

We further validated this two‐gene panel by performing feasible IHC experiments as follows (Fig. 5G,H): (a) Tumors were defined as luminal‐like if both *GATA3* and *AGR3*, or either one of them, were positive; and (b) tumors were defined as non‐luminal‐like only if they were profiled as negative *GATA3* and negative *AGR3*. In our IHC‐based cohort involving 81 Chinese histologic grade III ER^+^HER2^−^ patients, we found that non‐luminal‐like patients had larger tumor size, more metastatic lymph nodes, and worse DFS (multivariate HR non‐luminal‐like vs luminal‐like, 3.48, 95% CI, 1.17–10.42, *P* < 0.001) than those with luminal‐like tumors (Fig. 5J; Table S23).

## 4 Discussion

In this study, multi‐omics profiling of breast cancer tumors from six cohorts enriched with grade III ER^+^HER2^−^ patients was performed, portraying a distinctive patient subgroup that remained poorly characterized and underrepresented in previous genomic and molecular profiling studies [14, 15, 16]. Compared with low/intermediate histologic grade ER^+^HER2^−^ patients, grade III cases tended to have earlier age at diagnosis, larger tumors, greater risk of lymph node metastasis, and a higher chance of receiving chemotherapy.

Grade III ER^+^HER2^−^ patients had inferior survival outcomes compared to patients with grade I/II ER^+^HER2^−^, and future clinical trials are warranted to assess the predictive value of histologic grade. The landscapes of oncogenic alterations in grade III ER^+^HER2^−^ patients within benchmarked TCGA cohort bear different cancer driver events such as *TP53* and *ESR1*, compared with low/intermediate grade cases, and one of their most prevalent mutation signatures was associated with defective DNA double‐strand break‐repair by homologous recombination. Interestingly, genome‐wide DNA methylation profiling revealed certain hypomethylated loci in the promoter of *MKI67* within grade III ER^+^HER2^−^ patients compared to grade I/II tumors or normal tissues, leading to upregulating mRNA expression level of *MKI67*. Identification of focal amplifications of *CCND1*, *MYC*, and *MDM2*, representing potential candidate driver events in grade III ER^+^HER2^−^ tumors, was distinct from those of grade I/II tumors. Similarly, the GSEAs indicated that cell cycle and immune‐related factors were enriched in grade III ER^+^HER2^−^ tumors compared with counterparts with grade I/II tumors (Fig. S15). Dissecting the heterogeneity of intrinsic molecular subtypes within grade III ER^+^HER2^−^ cases, we found that patients with non‐luminal‐like tumors were associated with worse survival than those with luminal‐like. Furthermore, we developed a two‐protein IHC panel that reliably identified this high‐risk subgroup and demonstrated its association with increased levels of immune‐related signaling, such as CD8 effector T cells and dendritic cells (Fig. S16). Hence, high histologic grade ER^+^HER2^−^ patients appeared to harbor significant molecular differences from those with low/intermediate tumors that could hold important implications for patient stratification and treatment.

Several mechanisms of de novo and acquired endocrine therapy resistance have been described, including loss of ER expression, ER crosstalk with growth factor receptors, subclonal genomic alterations of tumor suppressors or drivers, and acquisition of *ESR1* fusions or activating *ESR1* missense mutations [23, 39, 40]. Our findings identified more acquired activating *ESR1* mutations within metastatic diseases, demonstrating that metastatic tumor cells with *ESR1* mutations are most frequently acquired under the aromatase inhibitor therapy [23]. In addition, targetable pathways were identified in *ESR1* mutant cells such as growth factor receptor (GFR), *PI3K*, cyclin‐dependent kinases (*CDK*) 2/7, and NOTCH signaling pathways [41], and clinical trials of inhibitors of these novel targets are urgently warranted. In addition, amplifications in *MYC* have been identified within primary and metastatic histologic grade III ER^+^HER2^−^ breast cancer and described as transcriptional regulator [23] with negative impact on survival. Pelicci *et al*. reported that deregulation of the *TP53‐MYC* axis in mammary tumors increased cancer stem cell content and plasticity and was a critical determinant of tumor growth and clinical aggressiveness [42], where *MYC* was a transcriptional target of *TP53* in mammary stem cells and was activated in breast tumors as a consequence of *TP53* loss, and similar findings were observed within TNBC [43, 44]. However, we found that *MYC* amplification was correlated with *TP53*‐independent cell cycle progression among patients with grade III ER^+^HER2^−^ tumors. FDA‐approved drug screen with in vivo validation thus provides a rationale for clinical evaluation of *MYC* inhibition, such as bortezomib in *MYC*‐driven neuroblastoma [45], and further experimental and clinical studies are warranted to validate efficacy of any *MYC* inhibition within ER^+^ or ER‐ breast cancer with *MYC* amplifications. Interestingly, those driver events (*MYC/MDM2* amplifications) were consistently enriched in deregulation of cell cycle signaling molecules, which were related to novel therapeutic targets, such as cyclin‐dependent kinase *(CDK) 4/6* inhibitors [46]. Three *CDK4/6* inhibitors (i.e., palbociclib, ribociclib, and abemaciclib), in combination with endocrine therapies as a first‐line therapy, demonstrated greater efficacy for ER^+^HER2^−^ metastatic breast cancer in postmenopausal women [47, 48, 49]. Although interruption of the senescence pathway by *MDM2* amplification within grade III ER^+^HER2^−^ tumors cases may cause resistance to *CDK4/6* inhibitors [46], CGM097, a *MDM2* inhibitor, showed synergistic effects in combination with *CDK4/6* inhibitors or fulvestrant, abrogating cells that are resistant to *CDK4/6* inhibitors [50]. Taken together, those results highlighted opportunities of optimizing endocrine therapy for grade III ER^+^HER2^−^ breast cancer patients.

In the current study, histologic grade III ER^+^HER2^−^ tumors were enriched in mutation signature S3 (homologous recombination deficiency, HRD) and harbored higher DNA damage response (DDR) score (Table S2) than low‐grade cases, indicating genomic instability within this subgroup, and the consequences of this finding could influence clinical practice. The poly (ADP‐ribose) polymerase (PARP) family members, notably *PARP1*, are key players in the repair of DNA single‐strand breaks [51]. It was well known that *PARP* inhibitors provided a significant benefit over standard therapy among patients within HER2‐negative metastatic breast cancer like endocrine‐resistant ER^+^ cases and a germline BRCA mutation in randomized phase III trials (OlympiAD and EMBRACA) [52, 53].

Our DNA methylation analyses integrated with mRNA data were somewhat inconsistent with the earlier study after considering molecular subtypes, where we found more hypomethylated CpGs in histologic grade III ER^+^HER2^−^ cases. Moreover, additionally upregulated genes such as *MMD2*, *RIPK2*, and *EIF3E* due to hypomethylated CpGs within grade III ER^+^HER2^−^ tumors also might interfere with endocrine therapy [54, 55, 56]. Recently, a study identified a hypomethylated ER‐positive breast cancer subtype presenting the best survival probability compared with the hypermethylated ER^+^ and hypomethylated ER‐negative subtypes, where certain upregulated genes like *SFRP1* and *WIF* have great potential to suppress the progression of ER^+^ breast cancer. Indeed, DNA methylation loss occurs frequently in cancer genomes [39, 57, 58]. A prior study indicated that a local CpG sequence context, termed solo‐WCGWs, was associated with preferential hypomethylation in partially methylated domains (PMD), where PMD hypomethylation depth correlated with somatic mutation density and cell cycle gene expression [59]. The hypomethylated loci identified within grade III ER^+^HER2^−^ tumors contributed to upregulated expression of cell cycle genes like *MKI67* (*cg18629132*), *CCND1* (*cg00347938*), and *CCNE2* (*cg05060175*), and those markers reflected the mitotic history of high‐differential tumors.

PAM50 intrinsic subtyping reveals tumor heterogeneity that may affect strategies of treatment regimens, thus identifying the discrepancy between IHC and intrinsic subtypes enables physicians to precisely tailor therapies [60, 61, 62, 63]. Our two‐gene (*GATA3* and *AGR3*) IHC‐based panel could classify the histologic grade III ER^+^HER2^−^ breast tumors into luminal‐like and non‐luminal‐like subtypes, for whom the benefit from endocrine therapy is limited. Expectedly, we found that the non‐luminal‐like subtype within high‐grade ER^+^HER2^−^ tumors had worse DFS. Besides our classifier, a nonluminal disease score [64] based on percentage of *ER*, *PR*, and *MKI67* tumor cells was easy, fast, and with the potential to be widely implemented to identify nonluminal disease within ER^+^/HER2^−^ breast cancer when gene expression data are not available. A recent study [65] presented *GATA3* and *MDM2* were synthetically lethal in ER^+^ breast cancer, where *MDM2* was a novel therapeutic target in *GATA3*‐deficient subsets. Those results support the usefulness of our two‐gene panel, in identifying a subgroup of ER^+^HER2^−^ breast cancer with bad prognosis as candidates for novel individualized therapy.

To our knowledge, this is the first comprehensive report of molecular characteristics of histologic grade III ER^+^HER2^−^. The multicenter patient‐based nature of this study offers a sufficient number of this rare phenotype with multi‐omics data and a long period of follow‐up to allow us to describe its clinical‐pathologic, genomic, epigenetic, transcriptomic, and intrinsic features.

### 4.1 Limitations

Several limitations of the present study need to be considered. First, the WCCCG cohort, a retrospective study, has inherent limitations when results are compared with randomized controlled trials. Second, neither FUSSC nor WCCCG cohort had gene expression data, and we failed to further validate the two‐gene IHC‐based panel via PAM50 intrinsic subtypes. Additionally, we did not have any cohort with neo‐adjuvant endocrine therapy to test predictive effect of histologic grade, where pathologic complete response rate is regarded as a surrogate endpoint for the evaluation of the efficacy of novel therapies or biomarkers.

## 5 Conclusions

This research provides timely evidence that inferior prognosis was more likely to occur in patients with high histologic grade ER^+^HER2^−^ tumors than counterparts with low/intermediate grade tumors, especially for nonluminal cases who could be identified using our two‐gene classifier in clinical practice. The findings from current study also highlight the importance of tailored therapy for histologic grade III ER^+^HER2^−^ breast cancers, and clinical trials are warranted to verify the potential targeted drugs that we mentioned.

## Conflict of interest

The authors declare no conflict of interest.

## Author contributions

KW, TX, JW, and GR conceived and designed the analysis. KW, LL, QL, SF‐E, and XZ collected the data. KW and XL conducted wet experiments. KW and SF‐E performed the bioinformatic analysis. KW and SF‐E wrote the paper. TF, HL, LB, JC, TX, JW, and GR modified the draft.

## Supporting information


**Fig. S1**. Differences between APOBEC enriched and nonenriched grade III ER^+^HER2^−^ samples.
**Fig. S2**. Lollipop plots of TP53 and PIK3CA for grade I/II ER^+^HER2^−^, grade III ER^+^HER2^−^ and TNBC from TCGA.
**Fig. S3**. Similarities of detected mutation signatures in grade III ER^+^HER2^−^ against validated signatures.
**Fig. S4**. A detailed overview of ELMER workflow.
**Fig. S5**. Volcano plot. Probes hypermethylated in grade III ER^+^HER2^−^ tumors vs grade I/II tumors (A) or normal solid tissue (B) and hypomethylated in grade III ER^+^HER2^−^ tumors vs normal solid tissue (C).
**Fig. S6**. Significant correlation analyses between hypomethylation loci level in grade III ER^+^HER2^−^ compared with grade I/II ER^+^HER2^−^ and mRNA expression levels by Pearson’s correlation test (all *P* < 0.05).
**Fig. S7**. Breast cancer‐specific survival analyses of ER^+^HER2^−^ tumors in METABRIC cohort according to MKI67 expression and histologic grade.
**Fig. S8**. GISTIC plots. Regions of gain A, C and loss B, D delineated for grade I/II ER^+^HER2^−^ (A and B) and nonmetastatic III ER^+^HER2^−^ (C and D) breast cancer by GISTIC analysis. Significance is reported as false discovery rate‐corrected *q*‐value
**Fig. S9**. Correlation between (A) MYC or (B) MDM2 gene expression level and copy number status within grade III ER^+^HER2^−^ tumors in METABRIC cohort.
**Fig. S10**. MYC amplification correlated with TP53‐independent cell cycle progression. (A) Expression levels of cell cycle‐related genes (CCNE2, MKI67) within grade III ER^+^HER2^−^ tumors in METABRIC cohort. (B) Correlation between MYC copy number status and TP53 mutation status within grade III ER^+^HER2^−^ tumors in METABRIC cohort.
**Fig. S11**. Genes’ selection strategy to identify the non‐luminal‐like subtype among grade III ER^+^HER2^−^ breast cancers.
**Fig. S12**. Correlation between the mRNA and protein expression of (A) GATA3, (B) AGR3 in the TCGA dataset.
**Fig. S13**. Receiver operating characteristic (ROC) curve as well as optimum cut‐off values of two genes (GATA3, AGR3) in predicting non‐luminal‐like subtypes within grade III ER^+^ HER2^−^ tumors in TCGA cohort and (A‐B) METABRIC cohort (C‐D).
**Fig. S14**. Comparison of breast cancer‐specific survival between luminal‐like subtype and non‐luminal‐like subtype that was inferred by two genes (GATA3, AGR3) among grade III ER^+^HER2^−^ cases (A) receiving endocrine therapy or (B) without endocrine therapy.
**Fig. S15**. GSEAs for grade III vs grade I/II ER^+^HER2^−^ tumors.
**Fig. S16**. Immune‐related GSVA score luminal‐like and non‐luminal‐like III ER^+^HER2^−^ tumors.
**Table S1**. Primers used in MSP.
**Table S2**. Data appendix to Fig. 2.
**Table S3**. Clinicopathological characteristics of grade III ER^+^ HER2^−^ breast cancer cases from SEER, METABRIC and TCGA cohorts compared with grade I/II ER^+^ HER2^−^ cases.
**Table S4**. Clinicopathological characteristics of grade III ER^+^ HER2^−^ breast cancer cases from MSKCC, WCCCG and FUSSC cohorts compared with grade I/II ER^+^ HER2^−^ cases.
**Table S5**. Univariate and multivariate analysis in disease‐free survival (DSF) by Cox proportional hazards models in WCCCG, FUSSC and MSKCC cohorts.
**Table S6**. Univariate and multivariate analysis in OS and breast cancer‐specific survival (BCSS) by Cox proportional hazards and Competing risk models in SEER, METABRIC and MSKCC cohorts.
**Table S7**. Breast cancer‐specific survival stratified by molecular subtypes by nuclear grade in the SEER dataset: 2010–2014.
**Table S8**. Hypomethylated probes and associated upregulated mRNA in grade III ER^+^HER2^−^ compared with grade I/II ER^+^HER^−^ cases identified by the ELMER package.
**Table S9**. Top 10 hypomethylated probes and associated upregulated mRNA in grade III ER^+^HER2^−^ compared with normal tissues identified by the ELMER package.
**Table S10**. Top 10 differential mutation events in grade I/II ER^+^HER2^−^, grade III ER^+^HER2^−^ and TNBC breast cancer from TCGA.
**Table S11**. Top 10 differential mutation events in grade I/II ER^+^HER2^−^, grade III ER^+^HER2^−^ and TNBC breast cancer from METABRIC.
**Table S12**. Top 10 differential mutation events in metastatic disease and primary tumors of patients with grade III ER^+^HER2^−^ from MSKCC.
**Table S13**. Top focal regions of amplification and deletion among grade III ER^+^HER2^−^ breast cancer cases from TCGA cohort.
**Table S14**. Top focal regions of amplification and deletion among metastatic grade III ER^+^HER2^−^ breast cancer cases from MSKCC cohort.
**Table S15**. Gene level CNA events from TCGA and METABRIC cohort.
**Table S16**. Gene level CNA events from MSKCC and METABRIC cohort.
**Table S17**. Univariate and multivariate analysis of amplification events by Cox proportional hazards models in grade III ER^+^HER2^−^ tumors from TCGA and METABRIC cohorts.
**Table S18**. Enriched pathways in grade III ER^+^HER2^−^ tumors with MYC amplification in C2 sets (curated sets) by GSEA (NOM *P* < 0.01, ES > 0.6).
**Table S19**. Enriched pathways in III ER^+^HER2^−^ tumors with MDM2 amplification in C2 sets (curated sets) by GSEA (NOM *P* < 0.01, ES > 0.7).
**Table S20**. Differential gene expression (DGE) in non‐luminal‐like compared with luminal‐like grade III ER^+^HER2^−^ cases.
**Table S21**. Differential pathways in nonluminal grade III ER^+^HER2^−^ tumors in C2 sets (curated sets) by GSVA (FDR < 0.05).
**Table S22**. Enriched pathways in nonluminal grade III ER^+^HER2^−^ tumors in C2 sets (curated sets) by GSEA (NOM *P* < 0.05).
**Table S23**. Clinicopathological characteristics of luminal‐like and non‐luminal‐like tumors within grade III ER^+^ HER2^−^ breast cancer patients of Chinese IHC‐based cohort.
**Appendix S1**. Study materials.

## Data Availability

The public data including TCGA, METABRIC, and MSKCC cohort that support the findings of this study are available in https://www.cbioportal.org/datasets. Two Chinese datasets, WCCCG and FUSSC, are available on request from the corresponding authors.
