# Supplementary material for: Integrated multi‐omics profiling of high‐grade estrogen receptor‐positive, HER2‐negative breast cancer
Source: Mol Oncol. 2021 Jul 29;16(12):2413–31. doi: 10.1002/1878-0261.13043 (PMC9208078; doi:10.1002/1878-0261.13043)
Supplement: Supplementary file 1 — Fig. S1. Differences between APOBEC enriched and nonenriched grade III ER+HER2− samples. Fig. S2. Lollipop plots of TP53 and PIK3CA for grade I/II ER+HER2−, grade III ER+HER2− and TNBC from TCGA. Fig. S3. Similarities of detected mutation signatures in grade III ER+HER2− against validated signatures. Fig. S4. A detailed overview of ELMER workflow. Fig. S5. Volcano plot. Probes hypermethylated in grade III ER+HER2− tumors vs grade I/II tumors (A) or normal solid tissue (B) and hypomethylated in grade III ER+HER2− tumors vs normal solid tissue (C). Fig. S6. Significant correlation analyses between hypomethylation loci level in grade III ER+HER2− compared with grade I/II ER+HER2− and mRNA expression levels by Pearson’s correlation test (all P < 0.05). Fig. S7. Breast cancer‐specific survival analyses of ER+HER2− tumors in METABRIC cohort according to MKI67 expression and histologic grade. Fig. S8. GISTIC plots. Regions of gain A, C and loss B, D delineated for grade I/II ER+HER2− (A and B) and nonmetastatic III ER+HER2− (C and D) breast cancer by GISTIC analysis. Significance is reported as false discovery rate‐corrected q‐value Fig. S9. Correlation between (A) MYC or (B) MDM2 gene expression level and copy number status within grade III ER+HER2− tumors in METABRIC cohort. Fig. S10. MYC amplification correlated with TP53‐independent cell cycle progression. (A) Expression levels of cell cycle‐related genes (CCNE2, MKI67) within grade III ER+HER2− tumors in METABRIC cohort. (B) Correlation between MYC copy number status and TP53 mutation status within grade III ER+HER2− tumors in METABRIC cohort. Fig. S11. Genes’ selection strategy to identify the non‐luminal‐like subtype among grade III ER+HER2− breast cancers. Fig. S12. Correlation between the mRNA and protein expression of (A) GATA3, (B) AGR3 in the TCGA dataset. Fig. S13. Receiver operating characteristic (ROC) curve as well as optimum cut‐off values of two genes (GATA3, AGR3) in predicting non‐luminal‐like subtypes [file MOL2-16-2413-s001.docx]

**ONLINE APPENDICES TO**

**Integrated Multi-omics Profiling of High-Grade Estrogen Receptor-Positive, HER2-Negative Breast Cancer**

***BY Kang Wang ET AL***

Contents

[Supplemental Methods 3](#_Toc74486140)

[Study Materials 3](#_Toc74486141)

[SEER cohort 3](#_Toc74486142)

[METABRIC cohort 3](#_Toc74486143)

[TCGA cohort 4](#_Toc74486144)

[MSKCC cohort 5](#_Toc74486145)

[WCCCG cohort 6](#_Toc74486146)

[FUSCC cohort 7](#_Toc74486147)

[Supplemental Results 9](#_Toc74486148)

[Supplemental Figures 9](#_Toc74486149)

[Figure S1 9](#_Toc74486150)

[Figure S2 10](#_Toc74486151)

[Figure S3 11](#_Toc74486152)

[Figure S4 12](#_Toc74486153)

[Figure S5 13](#_Toc74486154)

[Figure S6 14](#_Toc74486155)

[Figure S7 15](#_Toc74486156)

[Figure S8 16](#_Toc74486157)

[Figure S9 17](#_Toc74486158)

[Figure S10 17](#_Toc74486159)

[Figure S11 18](#_Toc74486160)

[Figure S12 19](#_Toc74486161)

[Figure S13 19](#_Toc74486162)

[Figure S14 20](#_Toc74486163)

[Supplemental Tables 22](#_Toc74486164)

[Table S1 22](#_Toc74486165)

[Table S2 23](#_Toc74486166)

[Table S3 24](#_Toc74486167)

[Table S4 26](#_Toc74486168)

[Table S5 28](#_Toc74486169)

[Table S6 29](#_Toc74486170)

[TableS7 30](#_Toc74486171)

[Table S8 31](#_Toc74486172)

[Table S9 31](#_Toc74486173)

[Table S10 32](#_Toc74486174)

[Table S11 32](#_Toc74486175)

[Table S12 33](#_Toc74486176)

[Table S13 33](#_Toc74486177)

[Table S14 34](#_Toc74486178)

[Table S15 35](#_Toc74486179)

[Table S16 36](#_Toc74486180)

[Table S17 36](#_Toc74486181)

[Table S18. 36](#_Toc74486182)

[Table S19 37](#_Toc74486183)

[Table S20 38](#_Toc74486184)

[Table S21 40](#_Toc74486185)

[Table S22 40](#_Toc74486186)

[Table S23 41](#_Toc74486187)

# Supplemental Methods

## Appendix S1. Study Materials

### SEER cohort

The five cohorts were included in this study. The first cohort, the Surveillance, Epidemiology, and End Results (SEER) database (<http://seer.cancer.gov/>) was used to assess the association between different subtypes ((i) grade III estrogen receptor (ER)+ human epidermal growth factor receptor 2 (HER2)-, (ii) grade I/II ER+HER2- , (iii) triple negative) and overall survival (OS)/breast cancer specific survival (BCSS) among (HER2) negative breast cancer patients who diagnosed from 2010 to 2014. To derive a dataset of patients with first primary invasive BC, the SEER*Stat software and ASCII files were used to access the SEER database. Female patients aged 18-80 years with HER2-negative, stage I-III, first primary breast cancer were comprised, and we excluded patients who were only initially identified by autopsy and death certificate, patients with bilateral tumor, and patients without information on ER/HER2 status and histological grade. Finally, we identified 150,060 eligible patients, which included 25,629 grade III ER+HER2- cases (17.1%).

### METABRIC cohort

METABRIC (Molecular Taxonomy of Breast Cancer International Consortium) is a Canada-UK project that aims to classify breast tumors into further subcategories, based on molecular signatures that will help determine the optimal course of treatment^1^. We downloaded its clinical and genomic data from cbioportal (<http://www.cbioportal.org/study?id=brca_metabric>), The molecular subtypes were determined by immunological histological chemistry (IHC) status supplied by METABRIC database, for exemple, ER positive was inferred by “ER_IHC” and “ER_status” positive, and HER2 negative was defined by “ER_status” after eliminating the “HER2_SNP6 gain”. The residual inclusion criteria is the same with that of SEER database. The METABRIC database included genomic data (copy number alteration (CNA) and somatic mutation data from targeted sequencing of 173 genes) as well as mRNA expression data (Illumina Human v3 microarray) where expression log intensity levels were employed. Lastly, we included 1,402 eligible patients, which included 404 grade III ER+HER2- cases (28.8%).

### TCGA cohort

The third cohort that we used is TCGA, whose clinical data was acquired from GDAC on December 30, 2016 (<http://gdac.broadinstitute.org/>) and complemented by The Pan-Cancer Atlas (PanCanAtlas) (https://gdc.cancer.gov/about-data/publications/pancanatlas) supplementary files. Although the information of nuclear grade in breast cancer was not primarily recorded in TCGA, we acquired this morphological feature data from previous works by Andrew et al ^2^. ER/PR/HER2 status was determined by IHC staining and fluorescence in situ hybridization (FISH) results: HER2 negative was defined as 1+/(0) or HER2 FISH status negative or ratio of HER2/CEP17 (ratio＜2.0) when HER2 IHC score was 2+. The TCGA Level 3 RNAseq Version 2 RSEM data, Level 3 WES data with tumor-specific mutations, somatic copy number alteration data composed genomic data, additionally, Reverse Phase Protein Array data and methylation (HM450 K) were also acquired from GDAC on December 30, 2016 (http://gdac.broadinstitute.org). The histological grade (a summary score of epithelial tubule formation, mitotic count and nuclear pleomorphism) data was retrieved based on a histological annotation for 850 invasive breast cases in TCGA, which was achieved by a team of 15 international breast cancer pathology experts (<https://onlinelibrary.wiley.com/doi/abs/10.1002/path.4847>) ^2^. The same inclusion and exclusion criteria before were used to selected patients, and this study included 508 HER2-negative breast cancer patients which comprised 88 (22.6%) grade III ER+HER2- cases. This study fully complies with the TCGA publication requirements (http://cancergenome.nih.gov/publications/publicationguidelines).

### MSKCC cohort

The fourth cohort form Memorial Sloan Kettering Cancer Center (MSKCC) (<http://www.cbioportal.org/study?id=breast_msk_2018>), a total of 1918 breast tumor specimens from 1756 patients, underwent prospective genomic profiling with return of results to patients and their physicians between April 2014 and March 2017^3^, which contained biopsy collection for treatment-naïve primary, post-treatment primary and metastatic tumor. The genomic data of patients in this study derived and confined within treatment-naïve primary tumors, whose histologic characteristics were determined based on clinical pathology reports. Treatment related data was acquired from supplementary files their recent study^3^, which recorded the types of treatments and detailed regimens. ER/PR status was defined as records called “ER/PR status of the primary”, and HER2 status was determined by “HER2 IHC Status Primary”, “HER2 IHC Score Primary”, “HER2 FISH Status (Report and ASCO) of Primary” and “HER2 FISH Ratio Primary”: HER2 negative was defined as 1+/(0) or HER2 FISH status negative or ratio of HER2/CEP17 (ratio＜2.0) when HER2 IHC score was 2+. Unlike before, we additionally included metastatic grade III ER+HER2- breast cancer patients, whose somatic copy number alterations (SCNA) data was used to explore the driven genes compared with that of non-metastatic grade III ER+HER2- cases. Finally, we included 590 non-metastatic HER2- cases comprising 272 (42%) grade III ER+HER2- patients, and 39 metastatic grade III ER+HER2- cases. To assess whether specific molecular alterations associated with endocrine treatment failure pre-existed therapy, we acquired and sequenced matched treatment-naive primary tumor specimens from a subset of affected patients.

### WCCCG cohort

The fifth cohort included in this study was obtained from Western China Clinical Cooperation Group (WCCCG), which included 23 breast cancer centers in nine provinces of Western China (i.e., Chongqing, Sichuan, Yunnan, Guizhou, Shanxi, Gansu, Guangxi, Ningxia and Xinjiang). The whole database included a total of 20,000 patients with breast cancer, which was histologically confirmed. Details about WCCCG had been described previously^4-6^. ER and PR positivity were determined by immunohistochemistry when the staining of ≥1 % of tumor cells appeared. Tumors were identified as HER-2-negative if they received an IHC score of 1+, and as HER2-positive only if they received an IHC score of 3+ or exhibited a HER2 gene expression level that was at least twofold higher than normal, which determined by FISH. We extracted the HER2- patients defined by inclusion criteria with completed survival data including survival status and survival time, who were followed up from 2005 to 2019, and questionnaire results were obtained through phone and the outpatient department follow-up ways. Patients in every registry would answer the questions through telephone follow-up or reexamine in outpatient department at least once every three months during the first three years and then every six months thereafter. Clinical doctors would take detailed history or have a completed physical examination at each follow-up visit. Residual breast ultrasound or mammogram, chest radiography, abdominal sonography, whole-body bone scan or PET/CT was routinely performed annually or when tumor relapse was clinically suspected. DFS was defined as the date of the diagnosis to the locoregional or distant recurrence or death from any cause, whichever came first, and DFS was considered as censored status if patients were alive until date of last contact. This observational study was entirely based on data extracted from patient medical records and was approved by the ethics committee of each participating center. We included 3783 HER2-negative breast cancer patients which comprised 546 (14.4%) grade III ER+HER2- cases.

### FUSCC cohort

The fifth cohort in this study was Fudan University Shanghai Cancer Center (FUSCC) cohort, whose Department of Breast Surgery included 2,502 post-operation HER2 negative breast cancer patients from January 1, 2012 to December 31, 2014. The patients were eligible if they met the prior inclusion criterial, which is the same with that of SEER cohort. Additionally, the clinicopathological and follow-up data in FUSSC cohort were determined and classified by the same way of WCCCG cohort. In total, there are 348 (13.9%) grade III ER+HER2- cases.

**References**:

1 C, C. *et al.* The genomic and transcriptomic architecture of 2,000 breast tumours reveals novel subgroups. *Nature* **486**, 346-352 (2012).

2 YJ, H. *et al.* The molecular basis of breast cancer pathological phenotypes. *The Journal of pathology* **241**, 375-391 (2017).

3 Razavi, P. *et al.* The Genomic Landscape of Endocrine-Resistant Advanced Breast Cancers. *Cancer Cell* **34**, 427-438 e426, doi:10.1016/j.ccell.2018.08.008 (2018).

4 K, W. *et al.* Comparison of Clinicopathological Features and Treatments between Young (≤40 Years) and Older (>40 Years) Female Breast Cancer Patients in West China: A Retrospective, Epidemiological, Multicenter, Case Only Study. *PloS one* **11**, e0152312 (2016).

5 K, W. *et al.* Clinicopathologic and Prognostic Significance of Body Mass Index (BMI) among Breast Cancer Patients in Western China: A Retrospective Multicenter Cohort Based on Western China Clinical Cooperation Group (WCCCG). *BioMed research international* **2019**, 3692093 (2019).

6 K, W. *et al.* Predictors of internal mammary lymph nodes (IMLN) metastasis and disease-free survival comparison between IMLN-positive and IMLN-negative breast cancer patients: Results from Western China Clinical Cooperation Group (WCCCG) database (CONSORT). *Medicine* **97**, e11296 (2018).

7 CH, M. *et al.* GISTIC2.0 facilitates sensitive and confident localization of the targets of focal somatic copy-number alteration in human cancers. **12**, R41, doi:10.1186/gb-2011-12-4-r41 (2011).

8 R, G. & bioinformatics, S. C. J. B. A flexible R package for nonnegative matrix factorization. **11**, 367, doi:10.1186/1471-2105-11-367 (2010).

9 Silva, T. C. *et al.* ELMER v.2: an R/Bioconductor package to reconstruct gene regulatory networks from DNA methylation and transcriptome profiles. *Bioinformatics* **35**, 1974-1977, doi:10.1093/bioinformatics/bty902 (2019).

10 A, C. *et al.* TCGAbiolinks: an R/Bioconductor package for integrative analysis of TCGA data. **44**, e71, doi:10.1093/nar/gkv1507 (2016).

11 Subramanian, A. *et al.* Gene set enrichment analysis: a knowledge-based approach for interpreting genome-wide expression profiles. *Proceedings of the National Academy of Sciences of the United States of America* **102**, 15545-15550, doi:10.1073/pnas.0506580102 (2005).

12 S, H., R, C. & bioinformatics, G. J. J. B. GSVA: gene set variation analysis for microarray and RNA-seq data. **14**, 7, doi:10.1186/1471-2105-14-7 (2013).

# Supplemental Results

## Supplemental Figures


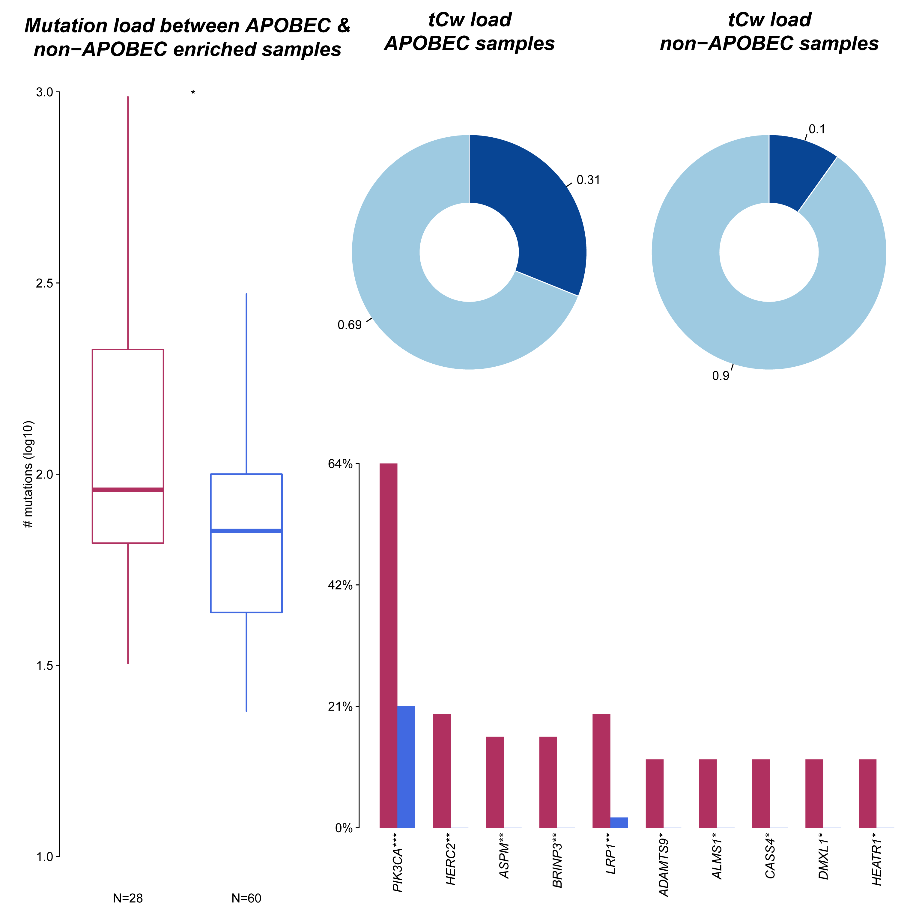


Figure S1 **Differences between APOBEC enriched and non-enriched grade III ER+HER2- samples.**


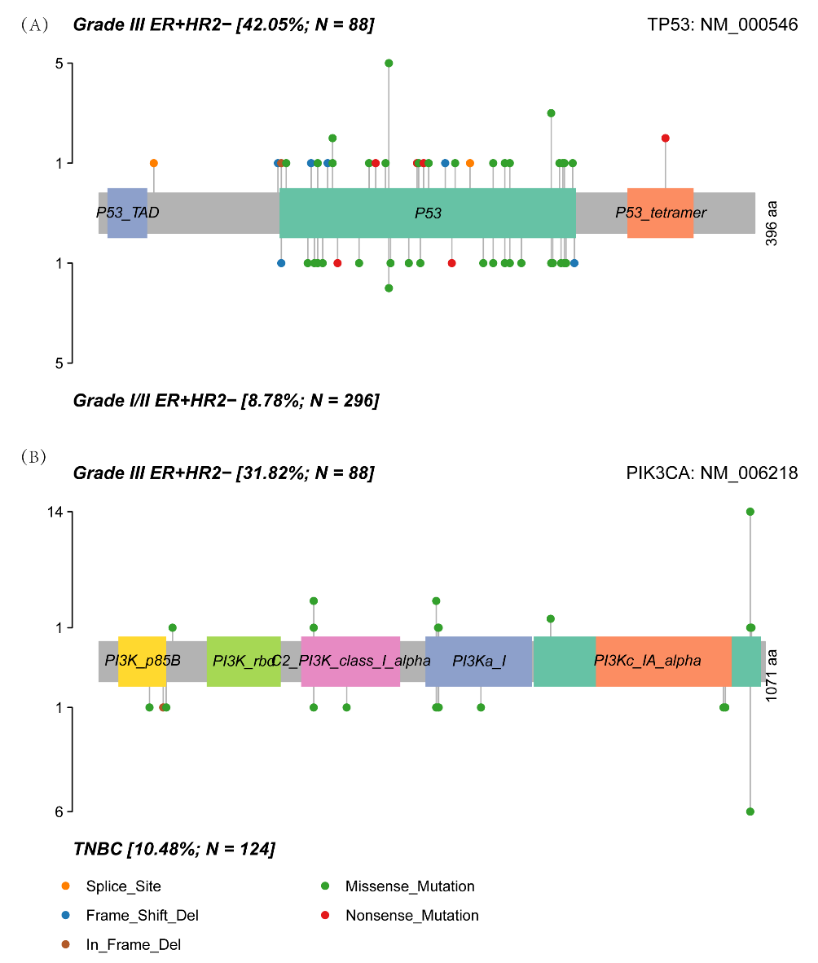


Figure S2 **Lollipop plots of TP53 and PIK3CA for grade I/II ER+HER2-, grade III ER+HER2- and TNBC from TCGA.**


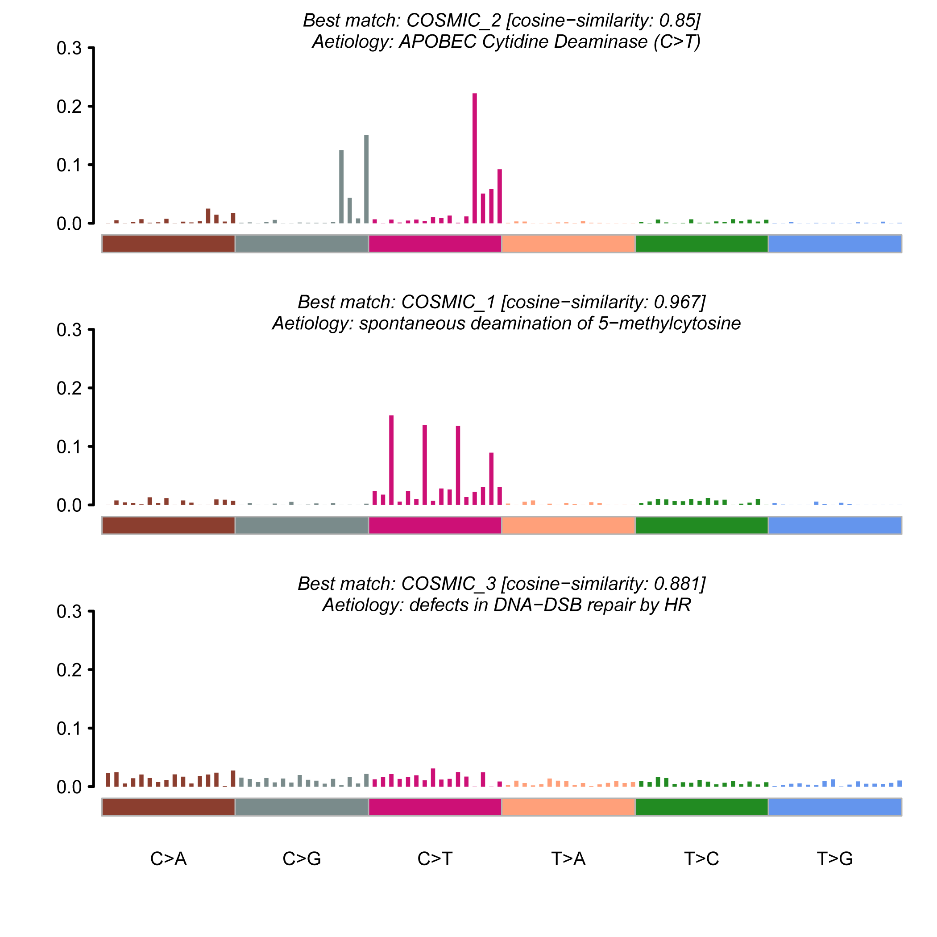


Figure S3 **Similarities of detected mutation signatures in grade III ER+HER2- against validated signatures**


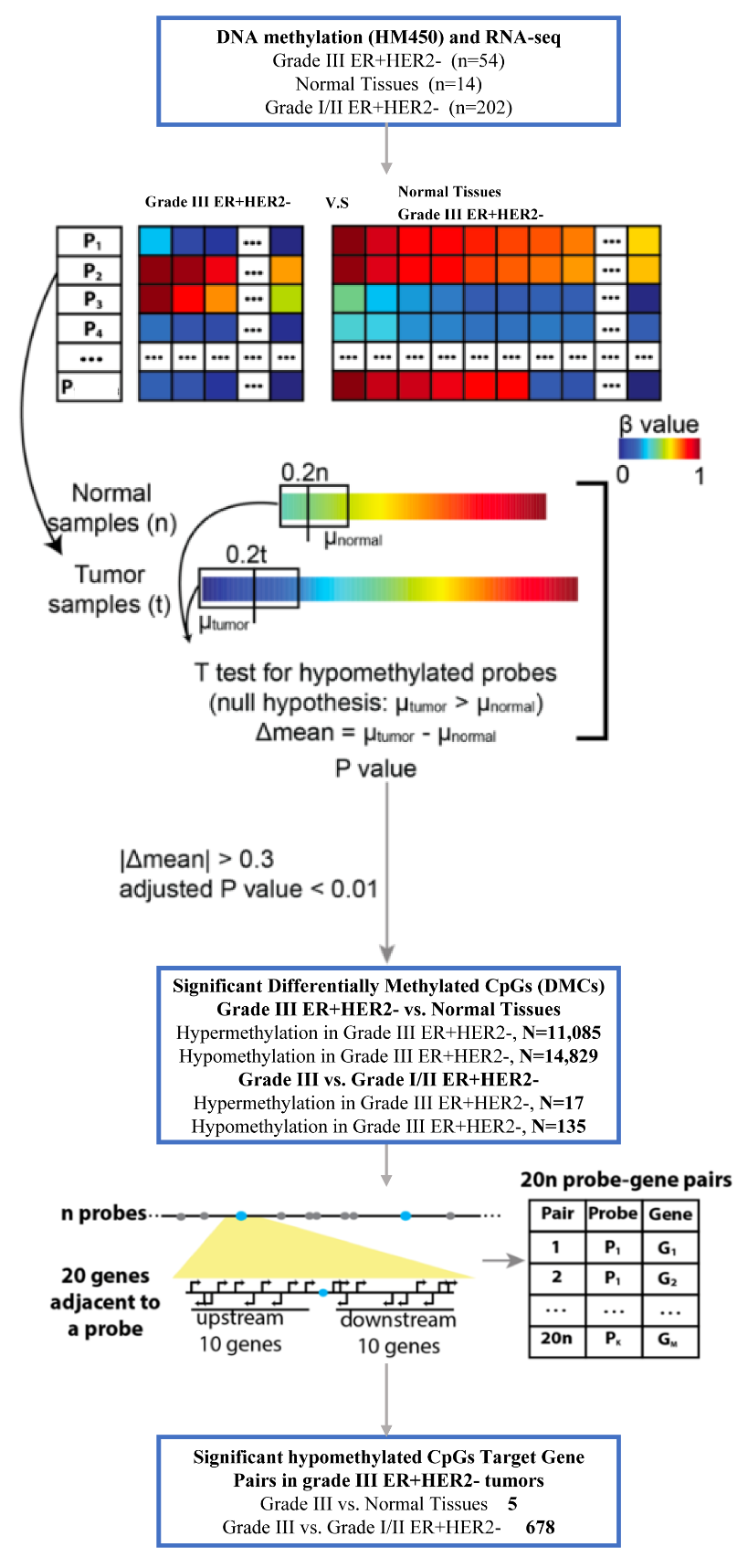


Figure S4**. A detailed overview of ELMER workflow (Ref:** <https://bioconductor.org/packages/release/bioc/vignettes/ELMER/inst/doc/index.html>**).**


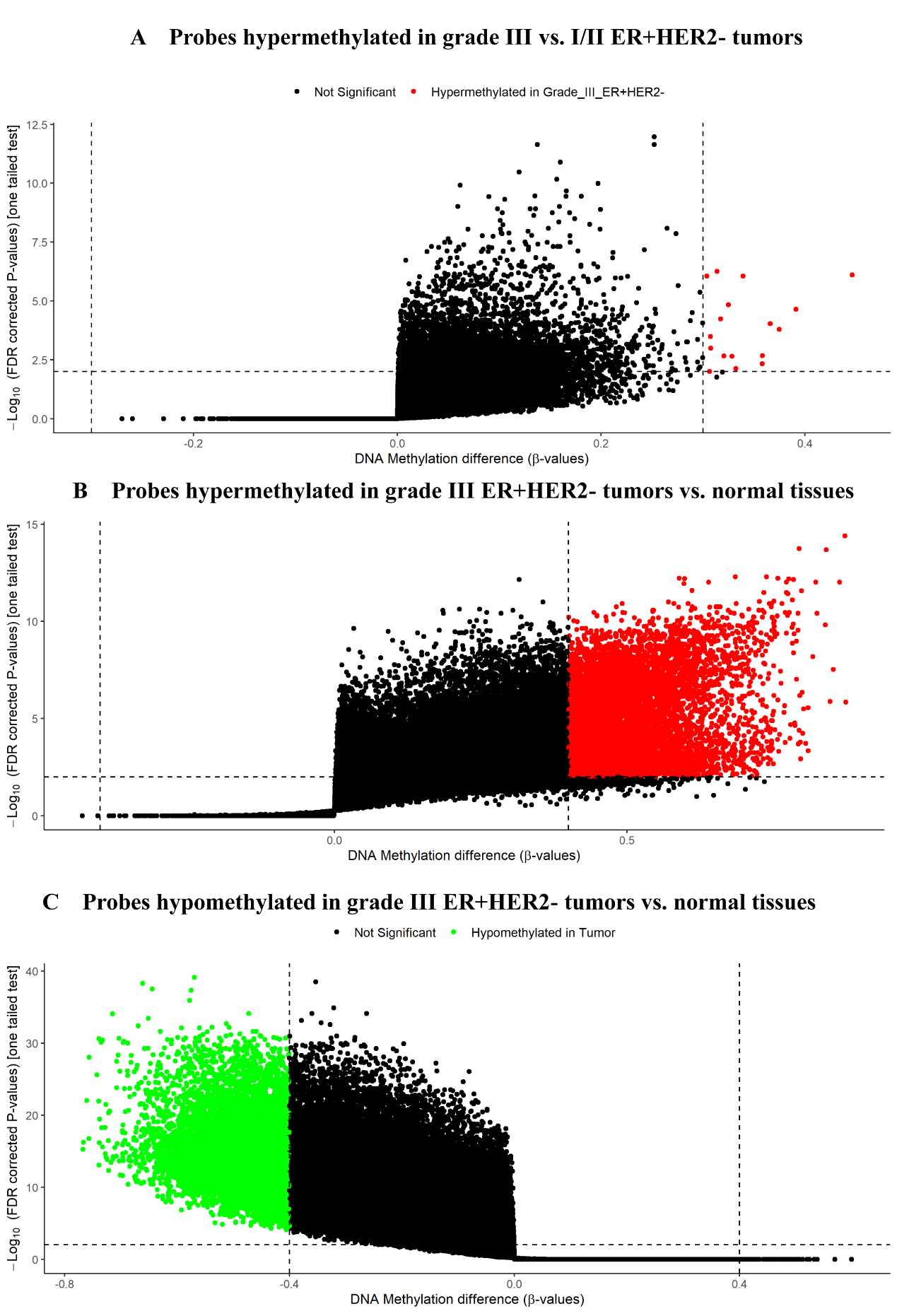


Figure S5 **Volcano plot. Probes hypermethylated in grade III ER+HER2- tumors vs grade I/II tumors (A) or normal** **solid tissue (B) and hypomethylated in grade III ER+HER2- tumors vs normal** **solid tissue (C).**


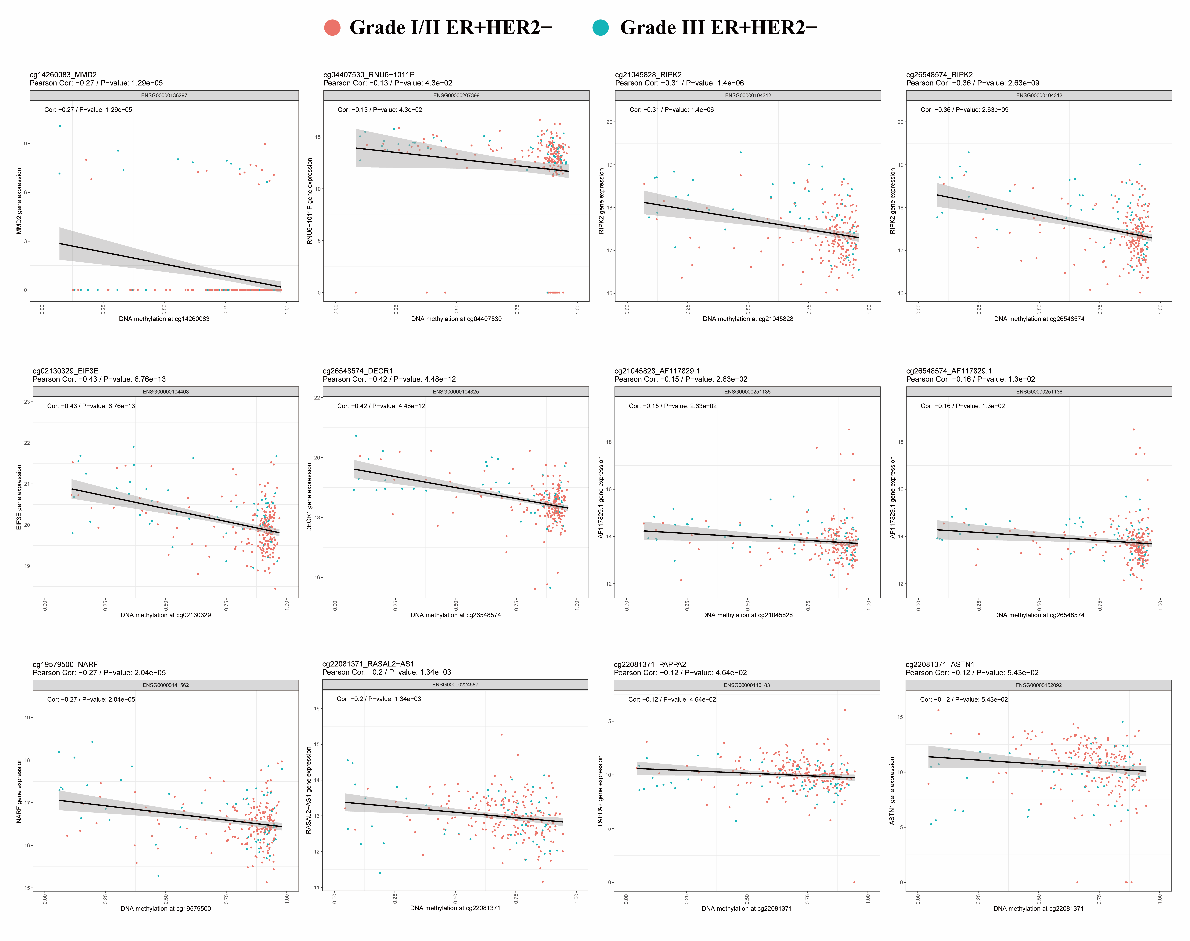


Figure S6 **Significant correlation analyses between hypo-methylation loci level in grade III ER+HER2- compared with grade I/II ER+HER2- and mRNA expression levels by Pearson’s**

**correlation test (all P<0.05).**

1. **(B)**


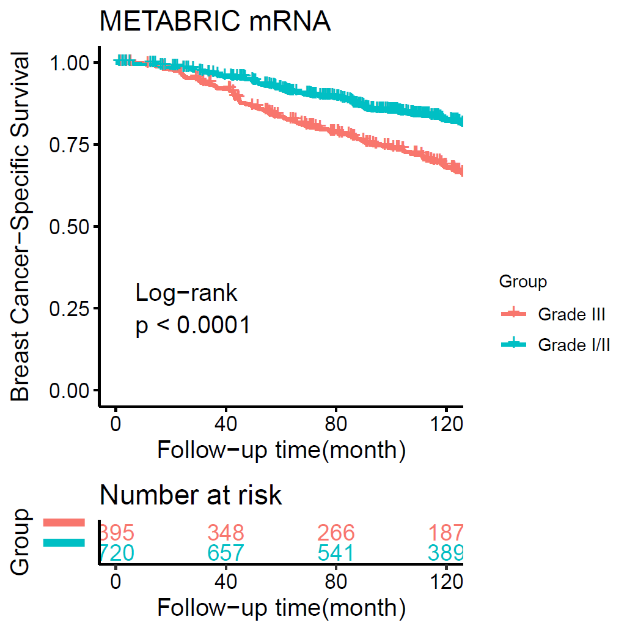

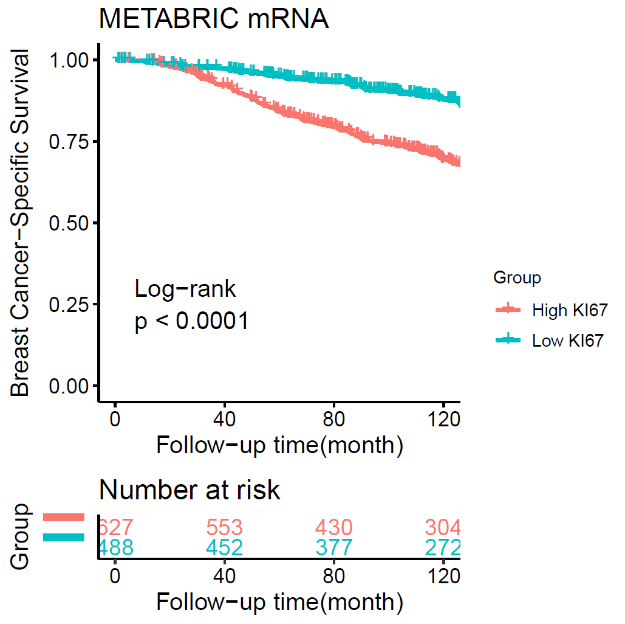


**(C)**


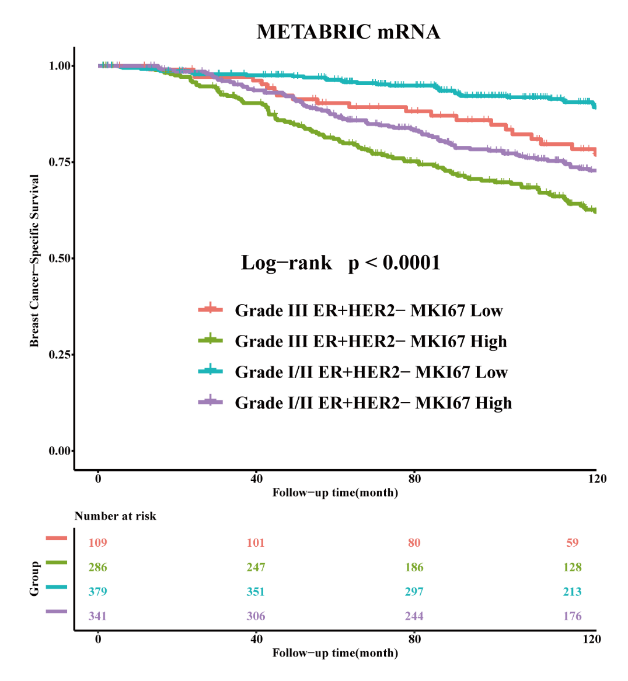


Figure S7**. Breast cancer-specific survival analyses of ER+HER2- tumors in METABRIC cohort according to MKI67 expression and histologic grade.**


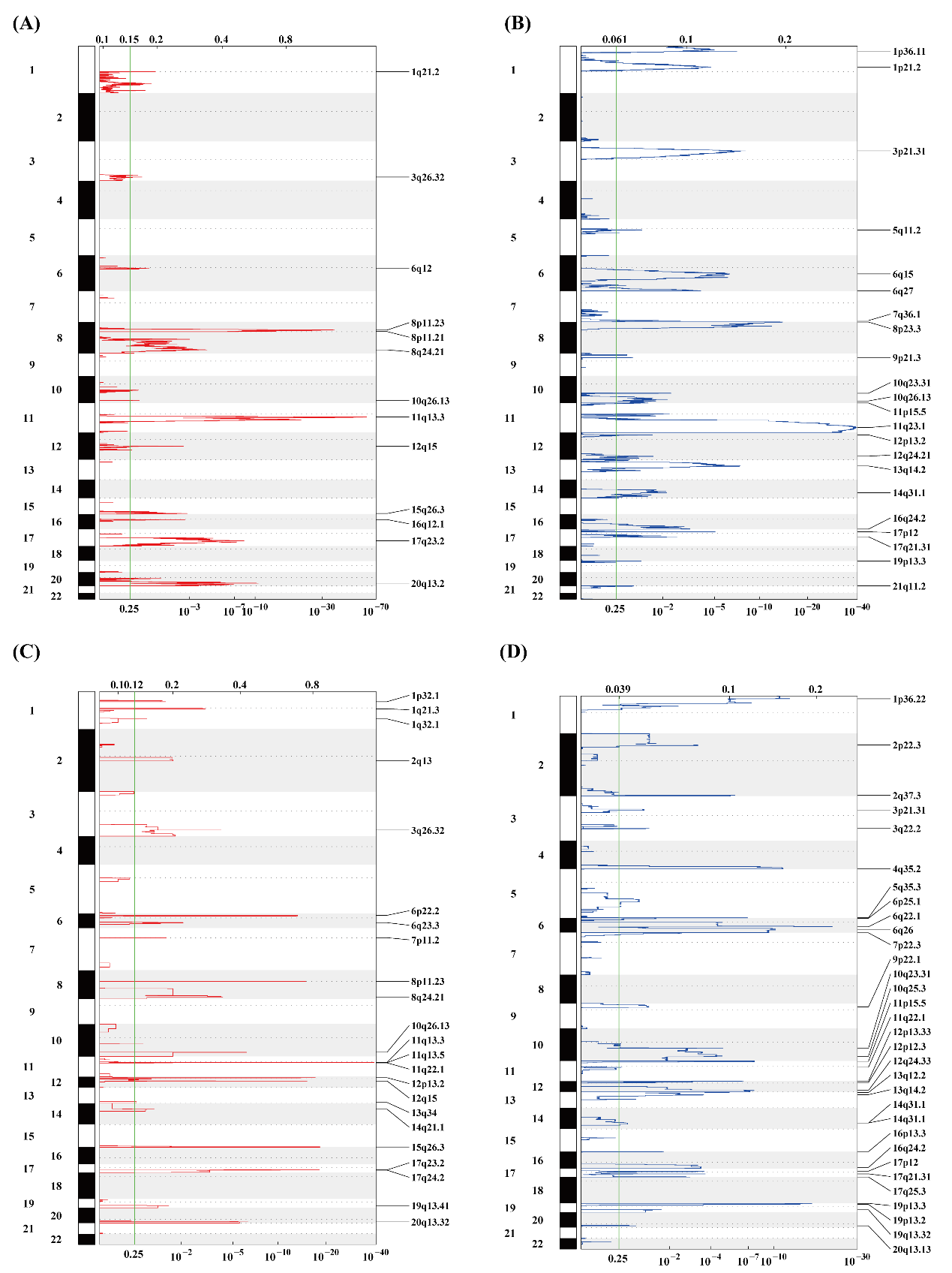


Figure S8 **GISTIC plots**. **Regions of gain A, C and loss B, D delineated for grade I/II ER+HER2- (A and B) and non-metastatic III ER+HER2- (C and D) breast cancer by GISTIC analysis. Significance is reported as false discovery rate-corrected q-value.**


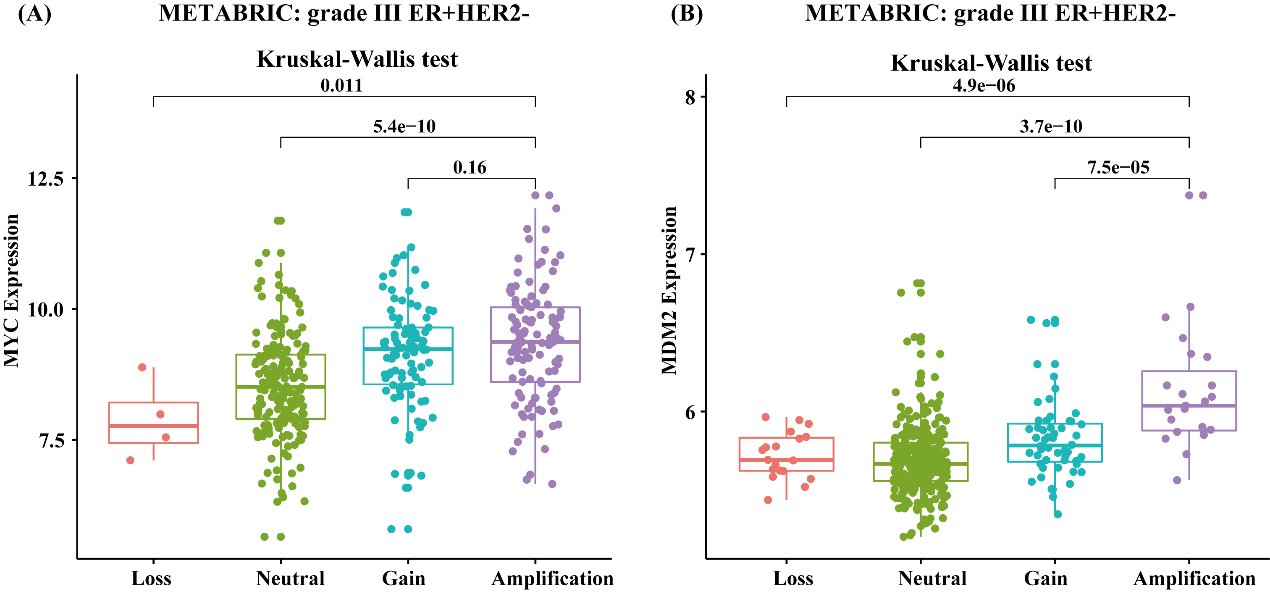


Figure S9**. Correlation between (A) MYC or (B) MDM2 gene expression level and copy number status within grade III ER+HER2- tumors in METABRIC cohort.**


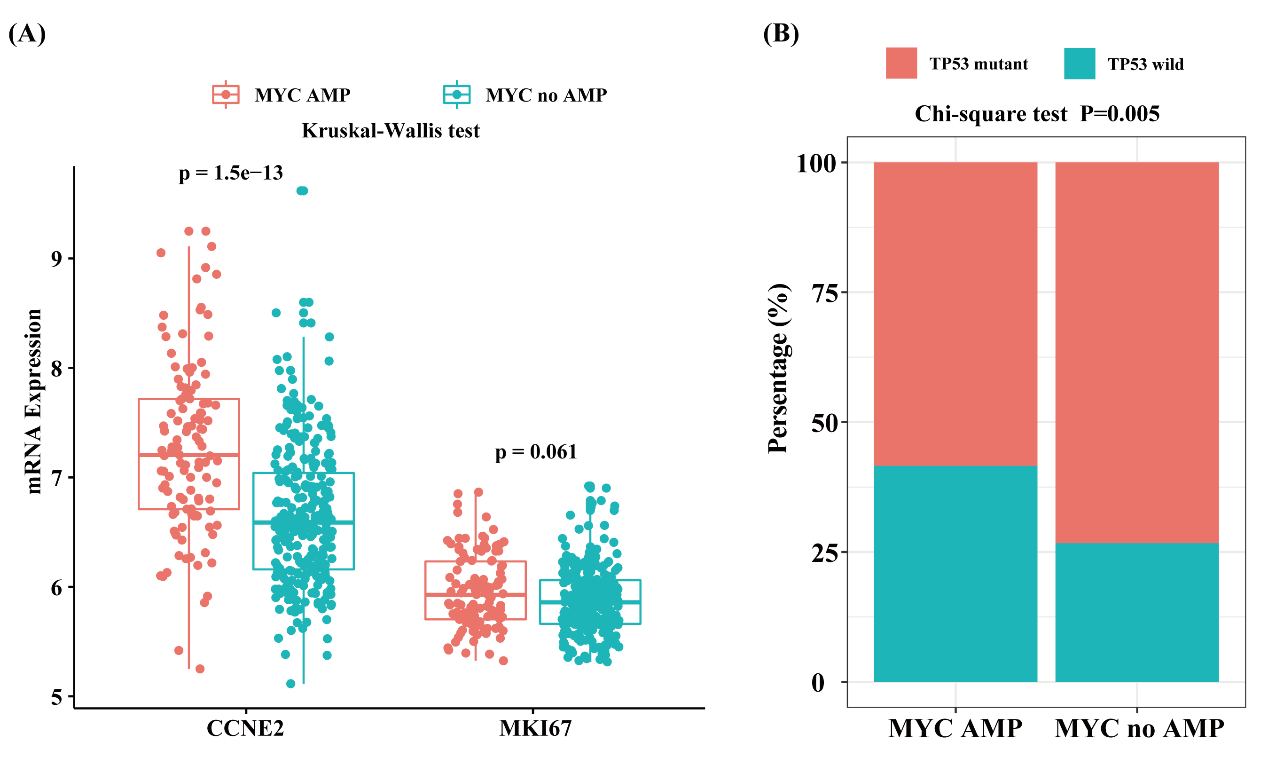


Figure S10**. MYC amplification correlated with TP53-independent cell-cycle progression**. **(A) Expression levels of cell-cycle related genes (CCNE2, MKI67) within grade III ER+HER2- tumors in METABRIC cohort. (B) Correlation between MYC copy number status and TP53 mutation status within grade III ER+HER2- tumors in METABRIC cohort.**


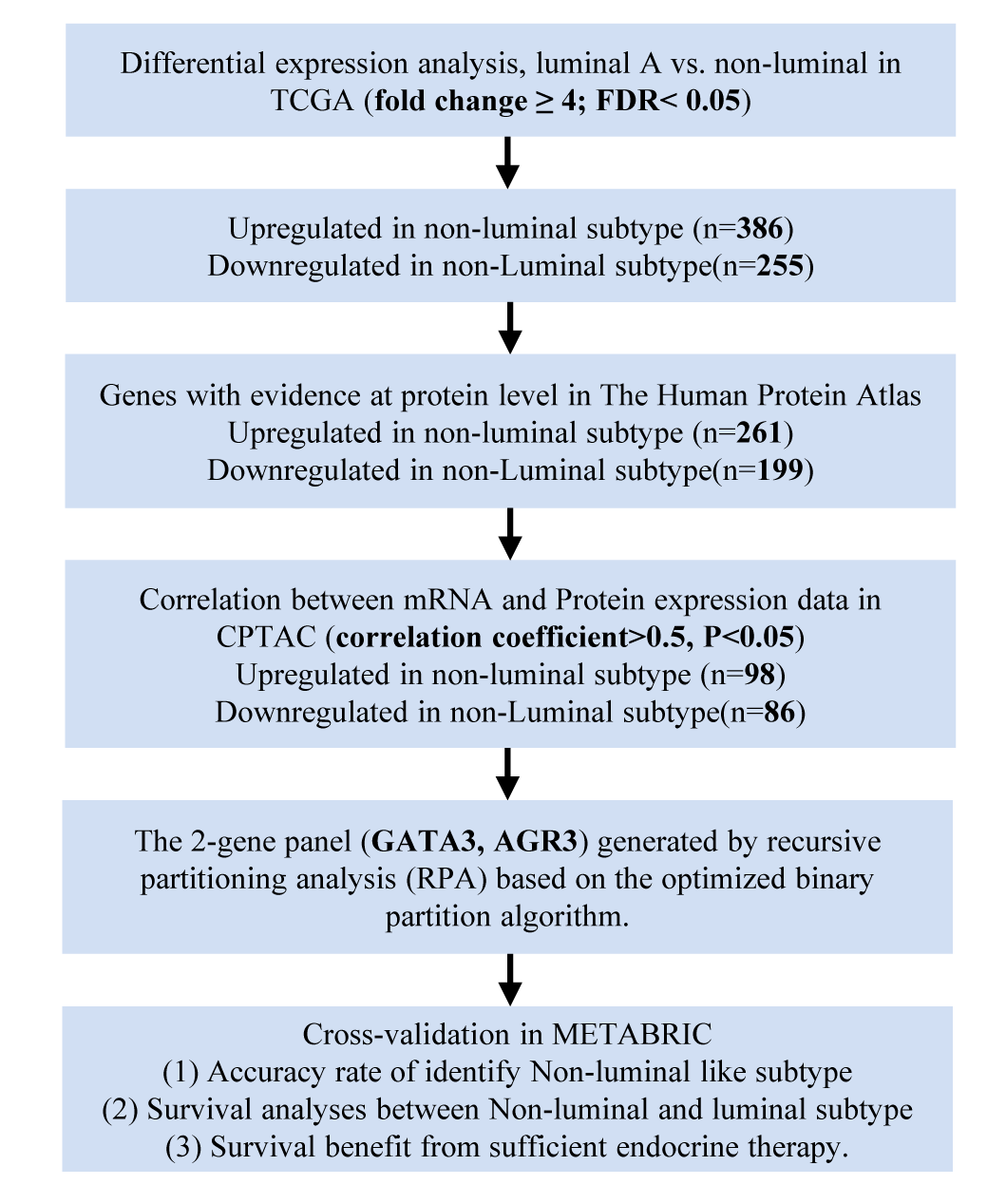


Figure S11**. Genes selection strategy to identify the Non-luminal like subtype among grade III ER+HER2- breast cancers.**


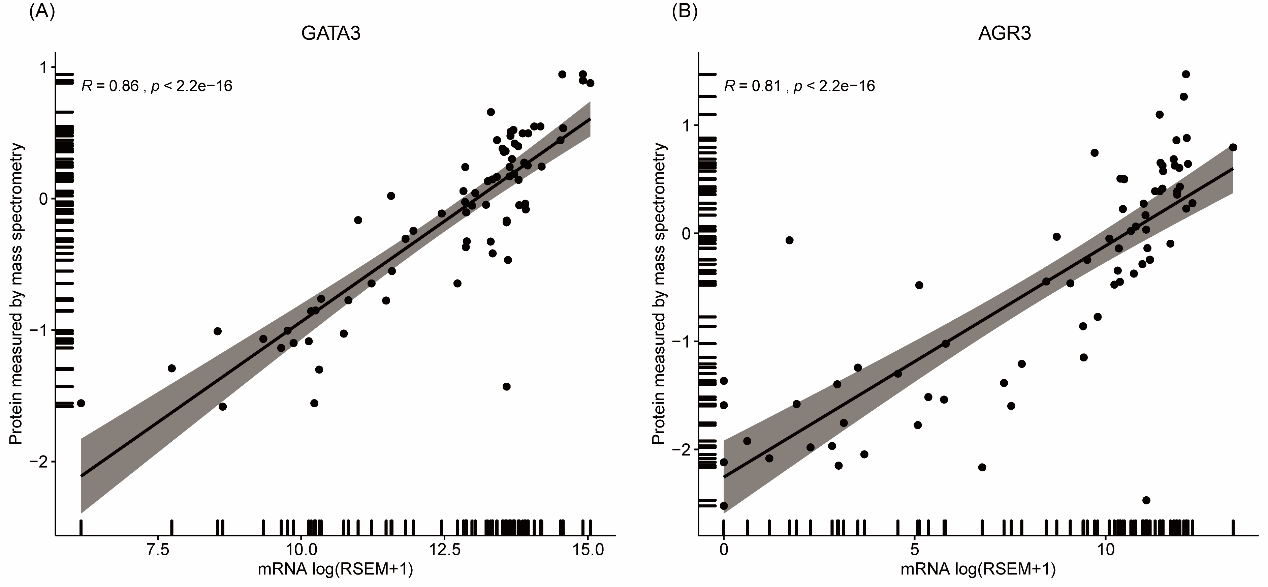


Figure S12**. Correlation between the mRNA and protein expression of (A) GATA3, (B) AGR3 in the TCGA dataset.**


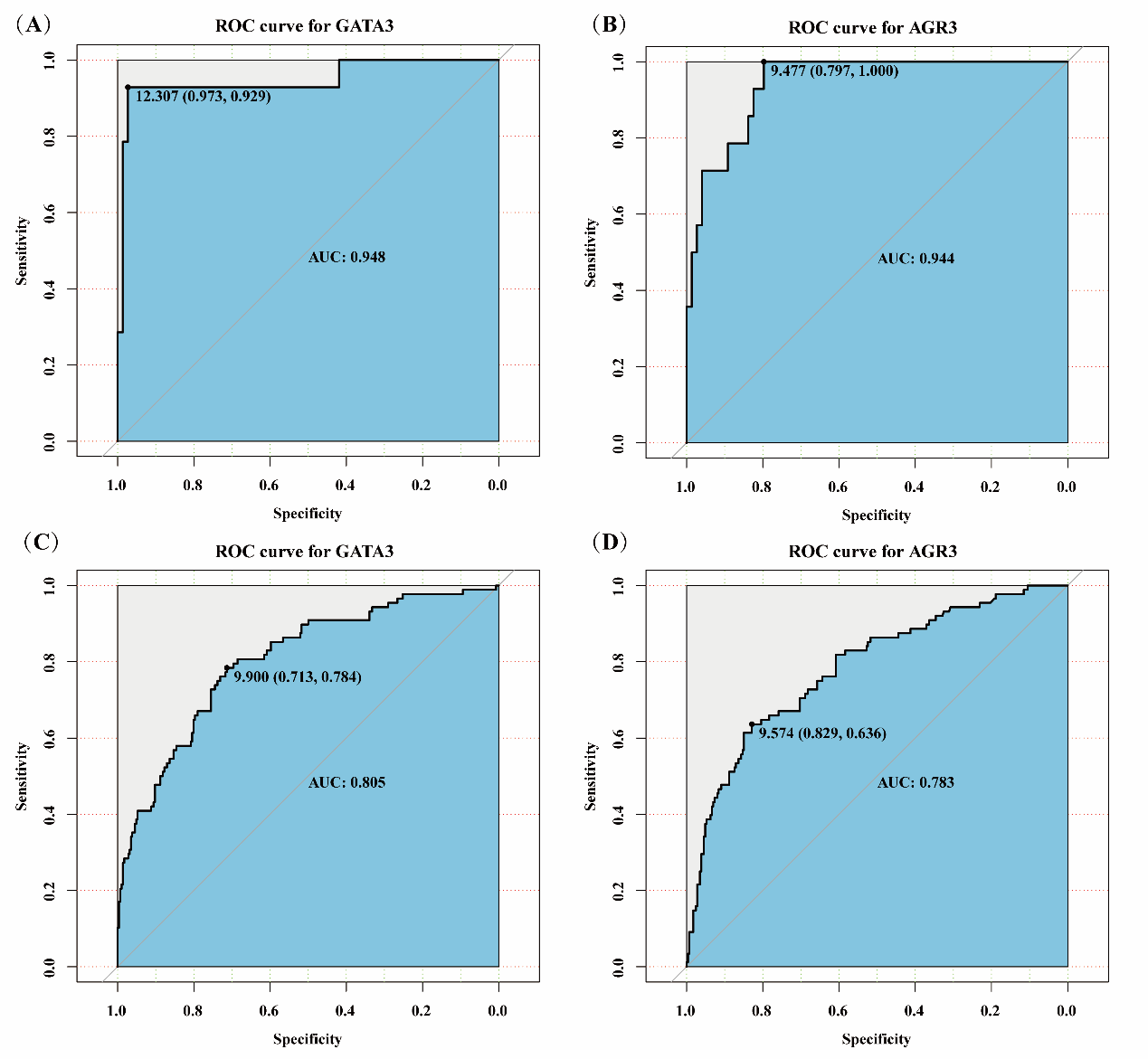


Figure S13 **Receiver operating characteristic (ROC) curve as well as optimum cut-off values of two genes (GATA3, AGR3) in predicting non-luminal-like subtypes within grade III ER+ HER2- tumors in TCGA cohort and (A-B) METABRIC cohort (C-D).**


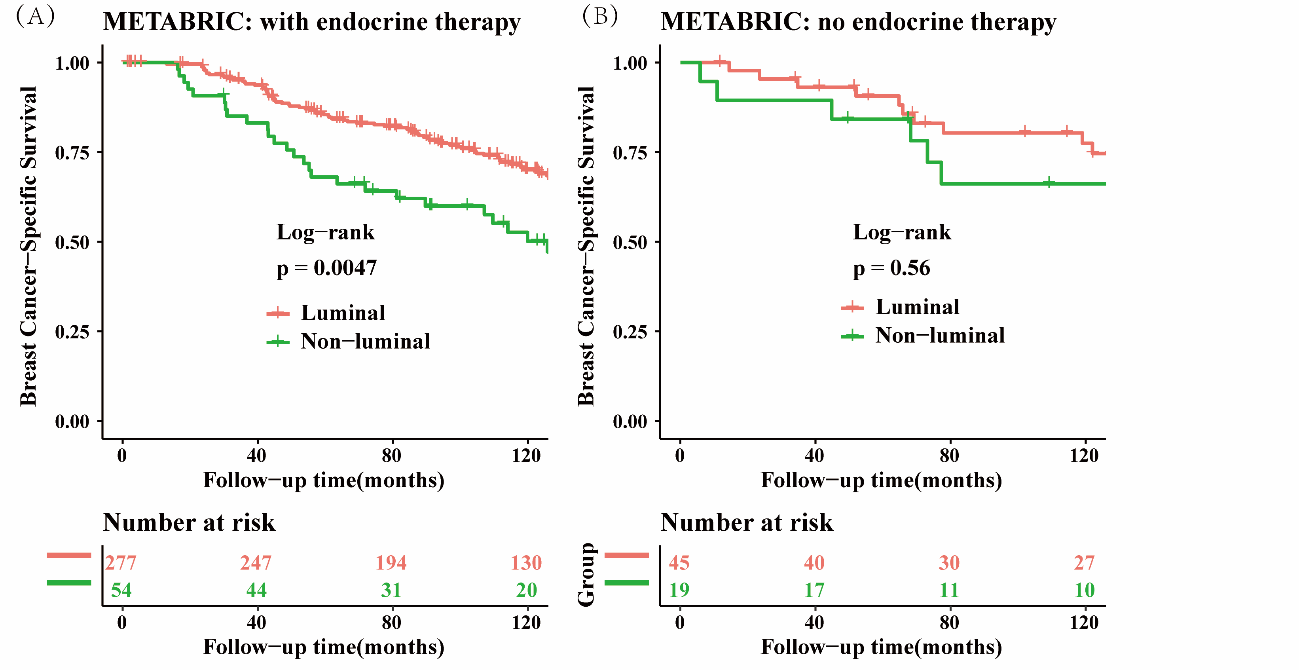


Figure S14**. Comparison of breast cancer-specific survival between luminal-like subtype and non-luminal like subtype that was inferred by two genes (GATA3, AGR3) among grade III ER+HER2- cases (A) receiving endocrine therapy or (B) without endocrine therapy.**


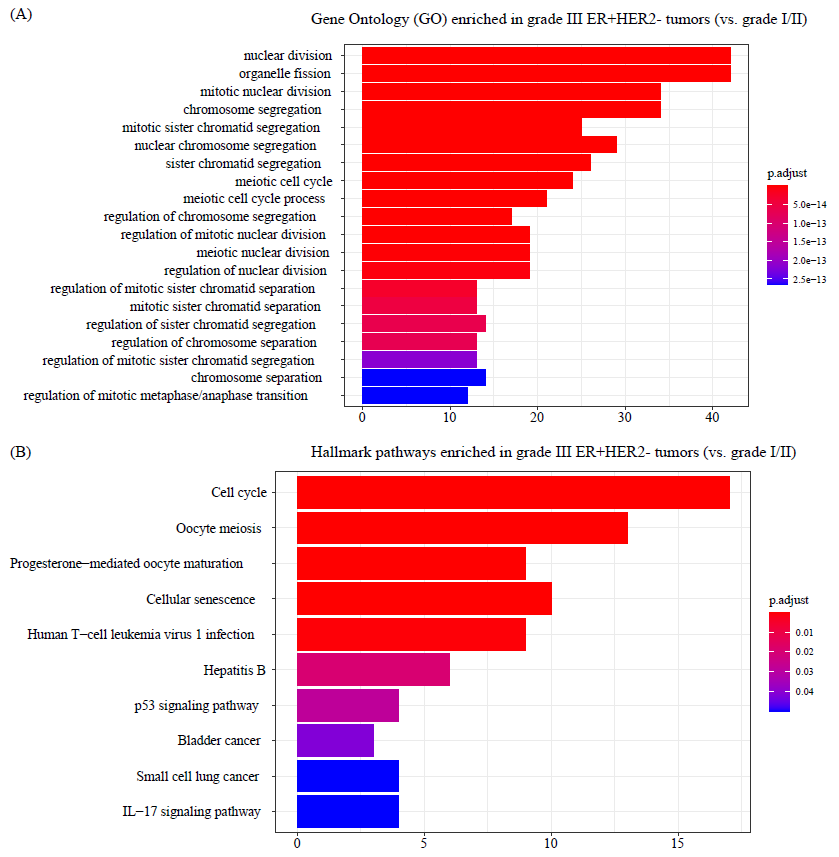


**Figure S15. GSEA analyses for grade III vs. grade I/II ER+HER2- tumors.**


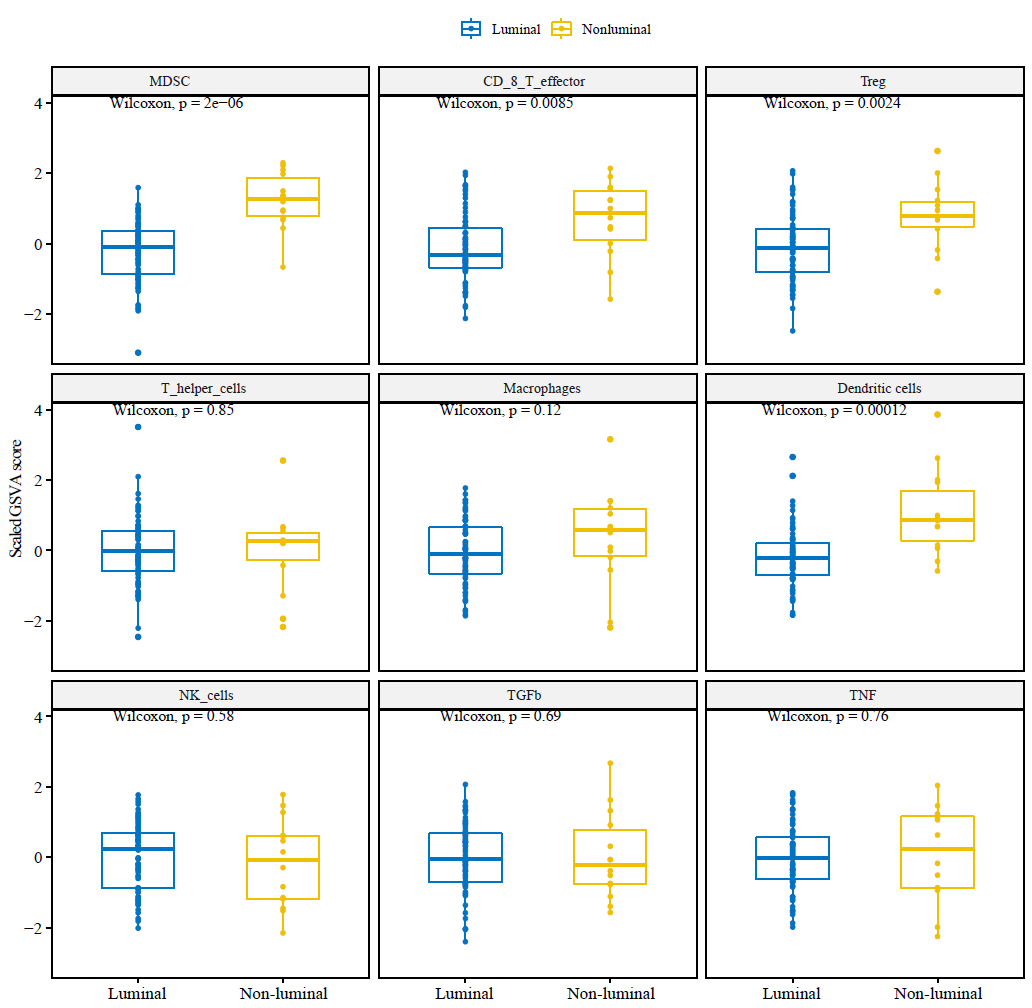


**Figure S16. Immune-related GSVA score luminal-like and non-luminal-like III ER+HER2- tumors.**

## Supplemental Tables

Table S1 **Primers used in methylation-specific PCR (MSP).**

| **Primer** | **Sequence (5'-3')** | **Product size (bp)** | **Annealing temp (℃)** |
| --- | --- | --- | --- |
| Ki67m1 | TTTGGCGGTAGAGGAGTAGTC | 122 | 60 |
| Ki67m2 | CGATCCTAAAAATACTTCATTAATT |  |  |
| Ki67u1 | GTTTGGTGGTAGAGGAGTAGTT | 123 | 58 |
| Ki67u2 | CAATCCTAAAAATACTTCATTAATT |  |  |
| Ki67F | AGCCTCAGCACCTGCTTGTT | 112 | 60 |
| Ki67R | ATATTGCCTCCTGCTCATGGA |  |  |

Table S2 **Data appendix to Figure 2.**

| **Molecular Features** | **Grade I/II ER+HER2-** | **Grade III ER+HER2-** | **TNBC** | **P** | |
| --- | --- | --- | --- | --- | --- |
|  |  |  |  | **Grade III ER+HER2-vs.**  **Grade I/II ER+HER2-** | **Grade III ER+HER2-vs. TNBC** |
| **Number of cases** | **N= 296 (58.3%)** | **N= 88 (17.3 %)** | **N= 124 (24.4%)** |  |  |
|  | **Mean (SD) ^a^** | **Mean (SD) ^a^** | **Mean (SD) ^a^** |  |  |
| **Tumor Purity** | 0.61 (0.18) | 0.63 (0.18) | 0.53 (0.20) | 0.4 | **0.001** |
| **Proliferation Score** | -0.44 (0.35) | -0.01 (0.37) | 0.25 (0.30) | **<0.001** | **<0.001** |
| **Cell Cycle Score** | -2.31 (1.43) | -0.82 (1.60) | 0.19 (1.91) | **<0.001** | **0.001** |
| **DNA Damage Response Score** | -1.26 (1.84) | -0.63 (1.60) | -0.28 (1.45) | **0.01** | 0.17 |
| **Hormone Score** | 5.89 (2.04) | 5.08 (2.49) | 0.17 (1.86) | **0.008** | **<0.001** |
| **PI3K/AKT Score** | -1.91 (2.83) | -1.62 (3.07) | 0.30 (3.22) | 0.47 | **<0.001** |
| **Ras/MAPK Score** | 1.62 (2.00) | 1.00 (1.80) | 1.50 (2.10) | **0.02** | 0.13 |
| **TSC/mTOR Score** | -0.42 (1.93) | 0.72 (2.41) | 0.95 (1.86) | **<0.001** | 0.51 |
| **Hormone Score** | **N (%)^b^** | **N (%)^b^** | **N (%)^b^** |  |  |
| **Pam50 Subtype** |  |  |  |  |  |
| Luminal A | 240 (81.1) | 28 (31.8) | 4 (3.2) | <0.001 | <0.001 |
| Luminal B | 47 (15.9) | 46 (52.3) | 1 (0.8) |  |  |
| HER2 Enrichment | 0 (0.0) | 2 (2.3) | 10 (8.1) |  |  |
| Basal | 0 (0.0) | 9 (10.2) | 99 (79.8) |  |  |
| Normal | 9 (3.0) | 3 (3.4) | 7 (5.6) |  |  |

^a^ P value was calculated from Wilcoxon Rank Sum test.

^b^ P value was calculated from Fisher’s exact.

Table S3 **Clinicopathological characteristics of grade III ER+ HER2- breast cancer cases from SEER, METABRIC and TCGA cohorts compared with grade I/II ER+ HER2- cases.**

|  | **SEER** | | | | **METABRIC** | | | | **TCGA** | | | |
| --- | --- | --- | --- | --- | --- | --- | --- | --- | --- | --- | --- | --- |
|  | **Grade I/II ER+HER2-** | **Grade III ER+HER2-** | **TNBC** | P-value^a^ | **Grade I/II ER+HER2-** | **Grade III ER+HER2-** | **TNBC** | P-value^a^ | **Grade I/II ER+HER2-** | **Grade III ER+HER2-** | **TNBC** | P-value^a^ |
|  | **N=104,157**  **(69.4%)** | **N=25,629 (17.1%)** | **N=20,274 (13.5%)** |  | **N= 749**  **(53.4%)** | **N= 404 (28.8%)** | **N= 249 (17.8%)** |  | **N= 296 (58.3%)** | **N= 88 (17.3 %)** | **N= 124 (24.4%)** |  |
| **Age (mean±SD)** | 59.65 (10.9) | 56.14 (11.9) | 55.66(12.0) | **<0.001** | 63.20 (12.0) | 63.39 (12.1) | 54.68 (13.7) | 0.81 | 59.31 (13.3) | 60.06 (13.7) | 55.49 (11.9) | 0.65 |
| **Histology Types** |  |  |  |  |  |  |  |  |  |  |  |  |
| IDC | 77,336 (74.2) | 22,378 (87.3) | 18,179 (89.7) | **<0.001** | 520 (69.4) | 351 (86.9) | 210 (84.3) | **<0.001** | 194 (65.5) | 78(88.6) | 110 (88.7) | **<0.001** |
| ILC | 13,676 (13.1) | 1,119 (4.4) | 196 (1.0) |  | 77 (10.3) | 22 (5.4) | 12 (4.8) |  | 75 (25.3) | 5 (5.7) | 4 (3.2) |  |
| Others | 13,145 (12.6) | 2,132 (8.3) | 1,899 (9.4) |  | 152 (20.3) | 31 (7.7) | 27 (10.8) |  | 27 (9.1) | 5 (5.7) | 9 (7.3) |  |
| Unknown | / | / | / |  | / | / | / |  | 0 (0.0) | 0 (0.0) | 1 (0.8) |  |
| **T stage** |  |  |  |  |  |  |  |  |  |  |  |  |
| T1 | 74,905 (71.9) | 11,522 (45.0) | 9,159 (45.2) | **<0.001** | 384 (51.3) | 143 (35.4) | 93 (37.3) | **<0.001** | 104 (35.1) | 16 (18.2) | 32 (25.8) | **0.005** |
| T2 | 23,899 (22.8) | 11,356 (44.3) | 8,704 (42.9) |  | 333 (44.5) | 236 (58.4) | 137 (55.0) |  | 146 (49.3) | 50 (56.8) | 79 (63.7) |  |
| T3-4 | 5,317 (5.1) | 2,721 (10.6) | 2,382 (11.7) |  | 26 (3.5) | 22 (5.4) | 18 (7.2) |  | 46 (15.5) | 22 (25.0) | 13 (10.5) |  |
| Unknown | 36 (0.1) | 30 (0.1) | 29 (0.1) |  | 6 (0.8) | 3 (0.7) | 1 (0.4) |  |  |  |  |  |
| **N stage** |  |  |  |  |  |  |  |  |  |  |  |  |
| N0 | 77,019 (73.8) | 14,334 (55.8) | 13,509 (66.5) | **<0.001** | 437 (58.3) | 196 (48.5) | 120 (48.2) | **0.002** | 99 (33.4) | 23 (26.1) | 53 (42.7) | 0.21 |
| N1-3 | 27,132 (26.0) | 11,290 (44.1) | 6,762 (33.4) |  | 309 (41.3) | 208 (51.5) | 128 (51.4) |  | 195 (65.9) | 63 (71.6) | 71 (57.3) |  |
| Unknown | 6 (0.1) | 5 (0.1) | 3 (0.1) |  | 3 (0.4) | 0 (0.0) | 1 (0.4) |  | 2 (0.7) | 2 (2.3) | 0 (0.0) |  |
| **TNM stage** |  |  |  |  |  |  |  |  |  |  |  |  |
| I | 62,815 (60.3) | 8,405 (32.8) | 7,460 (36.8) | **<0.001** | 352 (47.0) | 174 (43.1) | 97 (39.0) | **0.003** | 71 (24.0) | 10 (11.4) | 20 (16.1) | **0.02** |
| II | 32,984 (31.7) | 12,448 (48.6) | 9,657 (47.6) |  | 203 (27.1) | 138 (34.2) | 85 (34.1) |  | 161 (54.4) | 48 (54.5) | 79 (63.7) |  |
| III | 8,358 (8.0) | 4,776 (18.6) | 3,157 (15.6) |  | 20 (2.7) | 21 (5.2) | 14 (5.6) |  | 61 (20.6) | 28 (31.8) | 22 (17.7) |  |
| Unknown | / | / | / |  | / | / | / |  | 3 (1.0) | 2 (2.3) | 3 (2.4) |  |
| **Surgery** |  |  |  |  |  |  |  |  |  |  |  |  |
| BCS | 65,394 (62.8) | 13,129 (51.2) | 10,662 (52.6) | **<0.001** | 324 (43.3) | 156 (38.6) | 115 (46.2) | 0.28 | 76 (25.7) | 17 (19.3) | 45 (36.3) | **0.009** |
| Mastectomy | 38,763 (37.2) | 12,500 (48.8) | 9,612 (47.4) |  | 416 (55.5) | 244 (60.4) | 131 (52.6) |  | 140 (47.3) | 32 (36.4) | 54 (43.5) |  |
| Others and Unknown | / | / | / |  | / | / | / |  | 80 (27.0) | 39 (44.3) | 25 (20.2) |  |
| **Chemotherapy** |  |  |  |  |  |  |  |  |  |  |  |  |
| No/unknown | 77,122 (74.0) | 9,449 (36.9) | 3,976 (19.6) | **<0.001** | 702 (93.7) | 345 (85.4) | 103 (41.4) | **<0.001** | 211 (71.3) | 63 (71.6) | 35 (28.2) | 0.9 |
| Yes | 27,035 (26.0) | 16,180 (63.1) | 16,298 (80.4) |  | 47 (6.3) | 59 (14.6) | 146 (58.6) |  | 85 (28.7) | 25 (28.4) | 89 (71.8) |  |

Abbreviations: SD, standard deviation; IDC, invasive ductal carcinoma; ILC, invasive lobular carcinoma; BCS, breast conserving surgery; ER, estrogen receptor, HER2, human epidermal growth factor receptor 2, METABRIC, Molecular Taxonomy of Breast Cancer International Consortium; SEER, Surveillance, Epidemiology, and End Results; TCGA, The Cancer Genome Atlas database; TNBC: triple negative breast cancer.

^a^Pearson’s chi-square, Fisher’s exact and T test was performed between the III ER+ HER2- and I/II ER+ HER2- groups if needed.

Table S4 **Clinicopathological characteristics of grade III ER+ HER2- breast cancer cases from MSKCC, WCCCG and FUSSC cohorts compared with grade I/II ER+ HER2- cases.**

|  | **MSKCC**^a^ | | | | **WCCCG** | | | | **FUSCC** | | | |
| --- | --- | --- | --- | --- | --- | --- | --- | --- | --- | --- | --- | --- |
|  | **Grade I/II ER+HER2-** | **Grade III ER+HER2-** | **TNBC** | P-value^b^ | **Grade I/II ER+HER2-** | **Grade III ER+HER2-** | **TNBC** | P-value^b^ | **Grade I/II ER+HER2-** | **Grade III ER+HER2-** | **TNBC** | P-value^b^ |
|  | **N=259(%)** | **N=272(%)** | **N=64(%)** |  | **N= 1,648 (%)** | **N=** **546 (%)** | **N= 1,589 (%)** |  | **N= 1,601 (%)** | **N= 348 (%)** | **N= 553 (%)** |  |
| **Age (mean±SD)** | 57.52 (11.9) | 54.48 (12.3) | 55.54 (15.5) | **0.004** | 49.45 (14.60) | 48.33 (11.96) | 47.90 (13.50) | 0.1 | 52.49 (11.16) | 50.80 (11.03) | 50.09 (10.49) | **0.01** |
| **Histology Types** |  |  |  |  |  |  |  |  |  |  |  |  |
| IDC | 182 (70.3) | 223 (82.0) | 56 (87.5) | **0.006** | 1,579 (95.8) | 529 (96.9) | 1,370 (86.2) | **0.01** | 1544 (96.4) | 340 (97.7) | 543 (98.2) | 0.44 |
| ILC | 54 (20.8) | 33 (12.1) | 2 (3.1) |  | 10 (0.6) | 8 (1.5) | 17 (1.1) |  | 4 (0.2) | 1 (0.3) | 0 (0.0) |  |
| Others | 23 (8.9) | 16 (5.9) | 6 (9.4) |  | 59 (3.6) | 9 (1.6) | 202 (12.7) |  | 53 (3.3) | 7 (2.0) | 10 (1.8) |  |
| **T stage** |  |  |  |  |  |  |  |  |  |  |  |  |
| T1 | 204 (78.8) | 138 (50.7) | 37 (57.8) | **<0.001** | 631 (38.3) | 192 (35.2) | 361 (22.7) | **<0.01** | 1058 (66.1) | 185 (53.2) | 286 (51.7) | **<0.001** |
| T2 | 50 (19.3) | 104 (38.2) | 20 (31.2) |  | 653 (39.6) | 257 (47.1) | 610 (38.4) |  | 520 (32.5) | 159 (45.7) | 258 (46.7) |  |
| T3-4 | 5 (1.9) | 30 (11.0) | 7 (10.9) |  | 63 (3.8) | 45 (8.2) | 95 (6.0) |  | 17 (1.1) | 4 (1.1) | 5 (0.9) |  |
| Unknown | / | / | / |  | 301 (18.3) | 52 (9.5) | 523 (32.9) |  | 6 (0.4) | 0 (0.0) | 4 (0.7) |  |
| **N stage** |  |  |  |  |  |  |  |  |  |  |  |  |
| N0 | 193 (74.5) | 147 (54.0) | 44 (68.8) | **<0.001** | 817 (49.6) | 211 (38.6) | 684 (43.0) | **<0.01** | 986 (61.6) | 175 (50.3) | 379 (68.5) | **<0.001** |
| N1-3 | 66 (25.5) | 125 (46.0) | 20 (31.2) |  | 617 (37.4) | 306 (56.0) | 489 (30.8) |  | 604 (37.7) | 172 (49.4) | 171 (30.9) |  |
| Unknown | / | / | / |  | 214 (13.0) | 29 (5.3) | 416 (26.2) |  | 11 (0.7) | 1 (0.3) | 3 (0.5) |  |
| **TNM stage** |  |  |  |  |  |  |  |  |  |  |  |  |
| I | 182 (70.3) | 107 (39.3) | 31 (48.4) | **<0.001** | / | / | / |  |  |  |  |  |
| II | 62 (23.9) | 111 (40.8) | 21 (32.8) |  | / | / | / |  |  |  |  |  |
| III | 15 (5.8) | 54 (19.9) | 12 (18.8) |  | / | / | / |  |  |  |  |  |
| Unknown | / | / | / |  |  |  |  |  |  |  |  |  |
| **Surgery** |  |  |  |  |  |  |  |  |  |  |  |  |
| BCS | / | / | / |  | 110 (6.7) | 26 (4.8) | 106 (6.7) | **<0.01** | 353 (22.0) | 77 (22.1) | 126 (22.8) | 0.99 |
| Mastectomy | / | / | / |  | 1,069 (64.9) | 229 (41.9) | 1,101 (69.3) |  | 1248 (78.0) | 271 (77.9) | 427 (77.2) |  |
| Others and Unknown | / | / | / |  | 469 (28.5) | 291 (53.3) | 382 (24.0) |  |  |  |  |  |
| **Endocrine**  **therapy** |  |  |  |  |  |  |  |  |  |  |  |  |
| No/unknown | 76 (29.3) | 71 (26.1) | 62 (96.9) | 0.46 | 1299 (78.8) | 406 (74.4) | 1586 (99.8) | **0.04** | 154 (9.6) | 61 (17.5) | 536 (96.9) | **<0.001** |
| Yes | 183 (70.7) | 201 (73.9) | 2 (3.1) |  | 349 (21.2) | 140 (25.6) | 3 (0.2) |  | 1447 (90.4) | 287 (82.5) | 17 (3.1) |  |
| **Chemotherapy** |  |  |  |  |  |  |  |  |  |  |  |  |
| No/unknown | 181 (69.9) | 96 (35.3) | 9 (14.1) | **<0.001** | 231 (14.0) | 47 (8.6) | 172 (10.8) | **0.01** | 664 (41.5) | 46 (13.2) | 83 (15.0) | **<0.001** |
| Yes | 78 (30.1) | 176 (64.7) | 55 (85.9) |  | 1,417 (86.0) | 499 (91.4) | 1,417 (89.2) |  | 937 (58.5) | 302 (86.8) | 470 (85.0) |  |

Abbreviations: SD, standard deviation; IDC, invasive ductal carcinoma; ILC, invasive lobular carcinoma; BCS, breast conserving surgery; ER, estrogen receptor, HER2, human epidermal growth factor receptor 2, MSKCC, Memorial Sloan Kettering Cancer Center; WCCCG, Western China Clinical Cooperation Group; FUSCC, Fudan University Shanghai Cancer Center; TNBC: triple negative breast cancer.

^a^ Only I-III stage HER2- patients in MSKCC cohort were exhibited.

^b^Pearson’s chi-square, Fisher’s exact and T test was performed between the III ER+ HER2- and I/II ER+ HER2- groups if needed.

Table S5 **Univariate and multivariate analysis in disease-free survival (DSF) by Cox proportional hazards models in WCCCG, FUSSC and MSKCC cohorts.**

|  | **MSKCC**^c^ | | | | **WCCCG** | | | | **FUSSC** | | | |
| --- | --- | --- | --- | --- | --- | --- | --- | --- | --- | --- | --- | --- |
|  | **Grade I/II ER+HER2-** | **Grade III ER+HER2-** | **TNBC** | **P value^a^** | **Grade I/II ER+HER2-** | **Grade III ER+HER2-** | **TNBC** | **P value^a^** | **Grade I/II ER+HER2-** | **Grade III ER+HER2-** | **TNBC** | **P value^a^** |
| 5-year DSF rate (%) | 53.3% | 34.5% | 11.5% | **0.02** | 94.6% | 83% | 89.1% | **<0.001** | 95.7% | 81.8% | 87.5% | <0.001 |
| Univariate HR with 95% CI | Ref | **1.98**  **[1.19, 3.31]** | **2.33**  **[1.21, 4.50]** |  | Ref | **4.02**  **[2.26, 7.15]** | **2.49**  **[1.48, 4.20]** |  | Ref | **1.80**  **[1.16, 2.78]** | **2.86**  **[2.05, 4.00]** |  |
| Multivariate HR with 95% CI^b^ | Ref | **2.08**  **[1.22, 3.56]** | 2.05  [0.70, 6.05] |  | Ref | **2.52**  **[1.32, 4.81]** | **1.94**  **[1.08, 3.47]** |  | Ref | **1.56**  **[1.00, 2.44]** | **3.00**  **[1.64, 5.48]** |  |

Abbreviations: HR, hazard ratio; CI, confidence interval; DFS, disease-free survival; ER, estrogen receptor, HER2: human epidermal growth factor receptor 2; MSKCC, Memorial Sloan Kettering Cancer Center; WCCCG, Western China Clinical Cooperation Group; FUSCC, Fudan University Shanghai Cancer Center; TNBC, triple negative breast cancer.

^a^Log-rank tests were performed.

^b^Adjusted by Cox proportional hazards models including age, histology type, TNM stage, endocrine therapy, chemotherapy, and surgery.

^c^MSKCC cohort involved with univariate and multivariate analysis only included I-III stage HER2- patients.

Table S6 **Univariate and multivariate analysis in overall survival (OS) and breast cancer-specific survival (BCSS) by Cox proportional hazards and Competing risk models in SEER, METABRIC and MSKCC cohorts.**

|  | **SEER** | | | | **METABRIC** | | | | **MSKCC^d^** | | | |
| --- | --- | --- | --- | --- | --- | --- | --- | --- | --- | --- | --- | --- |
|  | **Grade I/II ER+HER2-** | **Grade III ER+HER2-** | **TNBC** | **P value^a^** | **Grade I/II ER+HER2-** | **Grade III ER+HER2-** | **TNBC** | **P value^a^** | **Grade I/II ER+HER2-** | **Grade III ER+HER2-** | **TNBC** | **P value^a^** |
| 5-year OS rate (%) | 94.2% | 86.9% | 80.4% | **<0.001** | 87.3 | 78.5% | 65.1% | **<0.001** | 82.3% | 80.4% | 29.9% | **<0.001** |
| Univariate HR with 95% CI | Ref | **2.33**  **[2.22, 2.44]** | **3.81**  **[3.65, 3.98]** |  | Ref | **1.38**  **[1.18, 1.62]** | 1.19  [0.98, 1.45] |  | Ref | 1.46  [0.65, 3.26] | **4.18**  **[1.7, 10.6]** |  |
| Multivariate HR with 95% CI^b^ | Ref | **1.95**  **[1.85, 2.04]** | **3.81**  **[3.63, 4.00]** |  | Ref | **1.20**  **[1.00, 1.45]** | 1.26  [0.97, 1.62] |  | Ref | 1.68  [0.74, 3.84] | 1.86  [0.47, 7.34] |  |
| 5-year BCSS rate (%) | 97.9% | 90.6% | 84.6% | **<0.001** | 91.7% | 83.4% | 67.5% | **<0.001** | / | / | / | / |
| Univariate HR with 95% CI | Ref | **4.78**  **[4.48,5.10]** | **8.52**  **[8.01, 9.06]** |  | Ref | **1.83**  **[1.48, 2.26]** | **2.18**  **[1.68, 2.84]** |  | / | / | / |  |
| Multivariate HR with 95% CI^c^ | Ref | **3.06**  **[2.85, 3.27]** | **6.28**  **[5.87, 6.71]** |  | Ref | **1.79**  **[1.44, 2.22]** | **2.24**  **[1.72, 2.93]** |  | / | / | / |  |

Abbreviations: OS, overall survival; BCSS, breast cancer-specific survival; HR, hazard ratio; CI, confidence internal; ER, estrogen receptor, HER2, human epidermal growth factor receptor 2, METABRIC, Molecular Taxonomy of Breast Cancer International Consortium; SEER, Surveillance, Epidemiology, and End Results; MSKCC, Memorial Sloan Kettering Cancer Center; TNBC: triple negative breast cancer.

^a^Log-rank tests were performed.

^b^Adjusted by Cox proportional hazards models including age, race, TNM stage, histology type, chemotherapy, and surgery.

^c^Adjusted by Competing risk models including age, race, TNM stage, histology type, chemotherapy, and surgery.

^d^MSKCC cohort involved with univariate and multivariate analysis only included I-III stage HER2- patients.

TableS7 **Breast cancer specific survival stratified by molecular subtypes by nuclear grade in the SEER dataset: 2010–2014.**

| **Molecular Subtypes** | **Grade I** | **Grade II** | **Grade III** | **P_trend_^a^** | **P_interaction_^b^** |
| --- | --- | --- | --- | --- | --- |
| **ER+HER2-(n=136,198)** |  |  |  |  | **<0.001** |
| 5-year BCSS rate (%) | 98.8 (98.7 to 99.0) | 96.8 (96.6 to 97.0) | 89.6 (89.2 to 90.1) |  |  |
| Multivariable-adjusted HR (95% CI)^c^ | 1.00 (reference) | **1.66(1.49 to 1.86)** | **4.31(3.85 to 4.82)** | **<0.001** |  |
| **ER+HER2+ (n=19,547)** |  |  |  |  |  |
| 5-year BCSS rate (%) | 98.2(97.3 to 99.1) | 95.8 (95.2 to 96.4) | 93.1 (92.5 to 93.7) |  |  |
| Multivariable-adjusted HR (95% CI)^c^ | 1.00 (reference) | **1.81 (1.12 to 2.94)** | **2.62 (1.63 to 4.23)** | **<0.001** |  |
| **HER2 enrichment (n=85,03)** |  |  |  |  |  |
| 5-year BCSS rate (%) | 89.0(81.7 to 96.9) | 91.8 (90.3 to 93.3) | 89.3(88.4 to 90.2) |  |  |
| Multivariable-adjusted HR (95% CI)^c^ | 1.00 (reference) | 0.88 (0.43 to 1.81) | 1.05 (0.52 to 2.11) | 0.13 |  |
| **TNBC (n=21,838)** |  |  |  |  |  |
| 5-year BCSS rate (%) | 94.8 (92.3 to 97.3) | 86.7(85.3 to 88.0) | 82.2 (81.5 to 82.8) |  |  |
| Multivariable-adjusted HR (95% CI)^c^ | 1.00 (reference) | **1.98 (1.22 to 3.23)** | **2.35 (1.46 to 3.80)** | **<0.001** |  |

^a^Linear trends across nuclear grade were tested by modeling the median value in each quintile as a continuous variable in Cox regression.

^b^P_interaction_ was calculated by adding the cross-product of tertile grade and molecular subtypes in the Multivariable-adjusted COX proportional hazards regression model.

^c^Adjusted by Cox proportional hazards models including age, race, TNM stage, histology type, chemotherapy, and surgery.

Table S8**. Hypomethylated probes and associated up-regulated mRNA in grade** **III ER+HER2- compared with grade I/II ER+HER- cases identified by the ELMER package.**

| **Probe** | **Ref Gene** | **△mean β value^a^** | **Adjusted P value** | **Raw.p^b^** | **Pe^c^** | **logFC^d^**  **(I/II vs. III)** | **FDR** |
| --- | --- | --- | --- | --- | --- | --- | --- |
| **cg18629132** | MKI67 | -0.41 | 4.34E-09 | 9.41E-08 | 0.001 | 1.15 | 2.49E-09 |
| cg14260083 | MMD2 | -0.35 | 2.28E-06 | 0.02 | 0.043 | 1.09 | 0.019 |
| cg04407530 | RNU6-1011P | -0.31 | 1.72E-06 | 6.77E-05 | 0.006 | 1.06 | 0.058 |
| cg21045828 | RIPK2 | -0.40 | 3.79E-09 | 0.0003 | 0.013 | 0.54 | 3.99E-07 |
| cg26548574 | RIPK2 | -0.43 | 2.05E-08 | 0.004 | 0.037 | 0.54 | 3.99E-07 |
| cg02130329 | EIF3E | -0.33 | 8.69E-06 | 0.0001 | 0.008 | 0.40 | 0.0002 |
| cg26548574 | DECR1 | -0.43 | 2.05E-08 | 0.0002 | 0.018 | 0.30 | 0.011 |
| cg21045828 | AF117829.1 | -0.40 | 3.79E-09 | 0.001 | 0.005 | 0.29 | 0.012 |
| cg26548574 | AF117829.1 | -0.43 | 2.05E-08 | 0.008 | 0.02 | 0.29 | 0.012 |
| cg19579500 | NARF | -0.39 | 4.28E-08 | 0.001 | 0.01 | 0.26 | 0.016 |
| cg22081371 | RASAL2-AS1 | -0.32 | 2.12E-07 | 0.007 | 0.04 | 0.03 | 0.787 |
| cg22081371 | PAPPA2 | -0.32 | 2.12E-07 | 0.007 | 0.009 | 0.56 | 0.007 |

^a^Differential DNA methylation level (mean β value) in III ER+HER2 vs. I/II ER+HER- samples.

^b^A number specify the raw p-value cutoff for defining significant pairs. It will select the significant P value cutoff before calculating the empirical p-values.

^c^A number specify the empirical p-value cutoff for defining significant pairs.

^d^Comparison of mRNA expression influenced by DNA methylation.

Abbreviations: logFC: log2 (fold change); FDR: false discovery rate.

Table S9**. Top 10 hypomethylated probes and associated up-regulated mRNA in grade III ER+HER2- compared with normal tissues identified by the ELMER package.**

| **Probe** | **Ref Gene** | **△mean β value^a^** | **Adjusted P value** | **Raw.p^b^** | **Pe^c^** | **logFC^d^**  **(III vs. normal)** | **FDR** |
| --- | --- | --- | --- | --- | --- | --- | --- |
| cg19214097 | AC079354.2 | -0.56 | 3.73E-14 | 1.26E-06 | 0.001 | **4.79** | **1.12E-15** |
| cg04742334 | AL023803.3 | -0.56 | 6.55E-13 | 1.53E-06 | 0.01 | **4.65** | **2.83E-14** |
| cg00735595 | AP000424.2 | -0.45 | 2.08E-20 | 2.39E-06 | 0.0002 | **4.41** | **5.28E-23** |
| cg01637789 | AC087878.1 | -0.57 | 1.01E-09 | 1.01E-07 | 0.01 | **4.31** | **1.07E-10** |
| cg18454510 | TDRG1 | -0.47 | 3.56E-17 | 1.17E-06 | 0.0009 | **4.31** | **4.01E-19** |
| cg16960046 | VSTM2A-OT1 | -0.51 | 3.18E-20 | 1.07E-06 | 0.001 | **4.29** | **8.99E-23** |
| cg12929160 | ACOD1 | -0.56 | 2.81E-13 | 3.30E-06 | 0.002 | **4.23** | **1.09E-14** |
| cg13320436 | AC060764.1 | -0.51 | 4.64E-20 | 1.07E-06 | 0.006 | **4.18** | **1.46E-22** |
| cg05707211 | AC124067.3 | -0.58 | 3.42E-18 | 6.18E-09 | 0.04 | **3.86** | **2.64E-20** |
| **cg18629132** | MKI67 | **-0.49** | **6.32E-17** | **7.82E-11** | **0.001** | **3.65** | **7.87E-19** |

^a^Differential DNA methylation level (mean β value) in III ER+HER2 vs. normal tissues.

^b^A number specify the raw p-value cutoff for defining significant pairs. It will select the significant P value cutoff before calculating the empirical p-values.

^c^A number specify the empirical p-value cutoff for defining significant pairs.

^d^Comparison of mRNA expression influenced by DNA methylation.

Abbreviations: logFC: log2 (fold change); FDR: false discovery rate.

Table S10 **Top 10 differential mutation events in** **grade I/II ER+HER2-, grade III ER+HER2- and TNBC breast cancer from TCGA.**

| **Differential Mutation Events** | **Grade III ER+HER2-** | **Grade I/II ER+HER2-** | **P^a^ value** | **Differential Mutation Events** | **Grade III ER+HER2-** | **TNBC** | **P^b^ value** |
| --- | --- | --- | --- | --- | --- | --- | --- |
| **TP53** | 37(42.0) | 26(8.8) | <0.001 | **TP53** | 37(42.0) | 93 (75.0) | <0.001 |
| **CSMD3** | 9(10.2) | 4(1.4) | <0.001 | **GATA3** | 13(14.8) | 0 | <0.001 |
| **BDP1** | 5(5.7) | 2(0.7) | 0.01 | **PIK3CA** | 28(31.2) | 13 (12.5) | 0.0002 |
| **MAGI1** | 5(5.7) | 2(0.7) | 0.01 | **MAP3K1** | 9(10.2) | 2(1.6) | 0.01 |
| **LRP1** | 6(6.8) | 4(1.4) | 0.01 | **GPR179** | 5(5.7) | 0 | 0.01 |
| **AKAP9** | 5(5.7) | 3(1.0) | 0.02 | **MAP2K4** | 5(5.7) | 0 | 0.01 |
| **CUBN** | 5(5.7) | 3(1.0) | 0.02 | **KMT2D** | 0 | 8(6.5) | 0.02 |
| **TNR** | 5(5.7) | 3(1.0) | 0.02 | **SI** | 0 | 8(6.5) | 0.02 |
| **DMD** | 6(6.8) | 5(1.7) | 0.02 | **IGSF10** | 0 | 6(4.8) | 0.04 |
| **TTN** | 18(20.5) | 32(10.8) | 0.03 | **ITPR2** | 0 | 6(4.8) | 0.04 |

^a^P value between grade I/II ER+HER2- and grade III ER+HER2- by chi-square test and Fisher’s exact test if needed.

^b^P value between III ER+HER2- and TNBC by chi-square test and Fisher’s exact test if needed.

Table S11 **Top 10 differential mutation events in grade I/II ER+HER2-, grade III ER+HER2- and TNBC breast cancer from METABRIC.**

| **Differential Mutation Events** | **Grade III ER+HER2-** | **Grade I/II ER+HER2-** | **P^a^ value** | **Differential Mutation Events** | **Grade III ER+HER2-** | **TNBC** | **P^b^ value** |
| --- | --- | --- | --- | --- | --- | --- | --- |
| **TP53** | 122 (30.2) | 82 (10.9) | <0.001 | **TP53** | 122 (30.2) | 199 (80.0) | <0.001 |
| **PIK3CA** | 154 (38.1) | 406 (54.2) | <0.001 | **PIK3CA** | 154 (38.1) | 39 (15.7) | <0.001 |
| **MAP3K1** | 33 (8.2) | 115 (15.4) | 0.01 | **GATA3** | 56 (13.9) | 3 (1.2) | <0.001 |
| **MYH9** | 23 (5.7) | 15 (2.0) | 0.01 | **CDH1** | 45 (11.1) | 9 (3.6) | 0.001 |
| **RB1** | 13 (3.2) | 6 (0.8) | 0.01 | **BRCA1** | 3 (0.7) | 13 (5.2) | 0.001 |
| **CBFB** | 16 (4.0) | 63 (8.4) | 0.02 | **ROS1** | 9 (2.2) | 17 (6.8) | 0.006 |
| **LAMB3** | 19 (4.7) | 14 (1.9) | 0.02 | **TBX3** | 19 (4.7) | 3 (1.2) | 0.01 |
| **ASXL2** | 14 (3.5) | 9 (1.2) | 0.02 | **MAP3K1** | 33 (8.1) | 9 (3.6) | 0.02 |
| **SPACA1** | 5 (1.2) | 1 (0.1) | 0.02 | **CTCF** | 13 (3.2) | 1 (0.4) | 0.02 |
| **COL22A1** | 24 (5.9) | 24 (3.2) | 0.03 | **CBFB** | 16 (4.0) | 2 (0.8) | 0.02 |

^a^P value between grade I/II ER+HER2- and grade III ER+HER2- by chi-square test and Fisher’s exact test if needed.

^b^P value between III ER+HER2- and TNBC by chi-square test and Fisher’s exact test if needed.

Table S12 **Top 10 differential mutation events in metastatic disease and** **primary tumors of patients with grade III ER+HER2- from MSKCC.**

| **Differential Mutation Events** | **Metastatic**  **Disease** | **Treatment Naive Primary** | **P^a^ value** | **OR**  **(odd ratios)** |
| --- | --- | --- | --- | --- |
| ESR1 | 25 (10.3) | 3 (1.1) | 2.59E-06 | 10.17 |
| TP53 | 118 (48.6) | 84 (31.1) | 6.60E-05 | 2.09 |
| KMT2C | 0 (0) | 11 (4.1) | 0.001 | 0 |
| MLL2 | 14 (5.8) | 3 (1.1) | 0.005 | 5.42 |
| APC | 11 (4.5) | 2 (0.7) | 0.009 | 6.33 |
| PLK2 | 11 (4.5) | 2 (0.7) | 0.009 | 6.33 |
| CDH1 | 18 (7.4) | 39 (14.4) | 0.01 | 0.47 |
| FAT1 | 18(7.4) | 7 (2.6) | 0.01 | 3.00 |
| ARID2 | 10 (4.1) | 2 (0.7) | 0.02 | 5.73 |
| ATR | 10 (4.1) | 2 (7.4) | 0.02 | 5.73 |

^a^P value between metastatic disease and primary tumors by chi-square test and Fisher’s exact test if needed.

Table S13 **Top focal regions of amplification and deletion among grade III ER+HER2- breast cancer cases from TCGA cohort.**

| **Cytoband^a^** | **q value** | **Peak region (Mb) ^a^** | **Number of genes^a,b^** | **Known proto-oncogene/ tumor suppressor gene in region^a,b^** |
| --- | --- | --- | --- | --- |
| Amplification | | | | |
| 8p11.23 | **1.55E-25** | 35.75 - 35.91 | 4 | **/** |
| 11q13.3 | **3.59E-13** | 66.04 - 66.35 | 3 | **CCND1** |
| 20q13.2 | **6.30E-07** | 49.29 - 50.12 | 3 | **/** |
| 8q24.21 | **1.32E-06** | 122.68 - 122.86 | 4 | **MYC** |
| 15q26.3 | **6.47E-06** | 94.48 - 94.94 | 3 | **/** |
| 12q15 | **3.15E-05** | 65.95 - 66.53 | 8 | **MDM2** |
| 17q23.1 | **4.21E-05** | 55.13 -55.63 | 14 | **/** |
| 19q12 | **0.036314** | 28.07 -29.54 | 11 | **CCNE1** |
| 19q13.42 | 0.062697 | 52.66 -53.71 | 54 | / |
| 6q25.1 | 0.10454 | 144.99 -145.72 | 2 | ESR1 |
| 14q13.1 | 0.11684 | 30.78 -32.55 | 6 | ARHGAP5 |
| 3p25.1 | 0.15635 | 13.84 -14.71 | 13 | / |
| 21q11.2 | 0.20112 | 0.01 -15.13 | 21 | / |
| 8q11.21 | 0.041688 | 40.94 - 67.67 | 105 | **PLAG1** |
| Deletion | | | | |
| 11q23.1 | **6.29E-06** | 96.31- 122.39 | 277 | **BCL9L** |
| 1p36.31 | **1.20E-05** | 0.00 - 8.52 | 146 | **SKI** |
| 8p23.2 | **2.16E-05** | 1.67 - 7.87 | 58 | **ARHGEF10** |
| 15q11.2 | **0.00050394** | 0.00 - 22.79 | 37 | **/** |
| 21q21.1 | **7.03E-05** | 17.13 - 21.10 | 11 | **/** |
| 6q21 | **0.0013043** | 103.55-104.61 | 7 | **/** |
| 17q21.31 | **0.0013043** | 40.38 - 42.77 | 52 | **/** |
| 19p13.3 | **0.0019567** | 0.00 - 3.46 | 134 | **STK11** |
| 22q11.1 | **0.0023041** | 0.00 - 16.92 | 14 | **/** |
| 7p22.3 | **0.0073183** | 0.00-10.47 | 95 | **CARD11** |
| 9p21.3 | **0.0013043** | 20.55-21.42 | 6 | **CDKN2A** |
| 2q37.3 | **0.0078784** | 231.05-231.93 | 15 | **/** |
| 12p12.3 | **0.0078784** | 11.84-15.29 | 40 | **CDKN1B** |
| 12q24.32 | **0.012311** | 73.87-127.65 | 428 | **TBX3** |
| 10q26.3 | **0.018979** | 128.84-129.26 | 21 | **/** |
| 17p12 | **0.019103** | 11.35-11.89 | 3 | **MAP2K4** |
| 3p13 | **0.029663** | 30.72-77.77 | 417 | **BAP1** |
| 11p15.4 | **0.049487** | 0.00-15.98 | 303 | **LMO1** |
| 7q36.1 | 0.067799 | 144.77-145.10 | 2 | / |
| 13q12.11 | 0.037884 | 0.00 - 18.84 | 3 | / |
| 5q12.3 | 0.11851 | 42.73-172.53 | 865 | MAP3K1 |
| 6p25.3 | 0.11851 | 0.00-11.46 | 78 | / |
| 14q31.1 | 0.12646 | 58.15-102.38 | 463 | AKT1 |
| 4q21.23 | 0.13981 | 71.03-136.33 | 307 | PTPN13 |
| 10p11.23 | 0.18858 | 29.48-30.14 | 1 | / |
| 21q11.2 | 0.00012901 | 0.00-14.76 | 16 | / |
| 9q21.11 | 0.11851 | 36.14-80.30 | 103 | GNAQ |
| 13q14.2 | 0.025581 | 0.00-109.83 | 414 | BRCA2 |

^a^Based on hg19 human genome assembly.

^b^RefSeq genes only.

^c^Known tumor suppressor genes and proto-oncogenes defined as found in COSMIC; if there is more than one known proto-oncogene in the region, only one is listed (priority for listing is, in order: known breast mutation; other known mutation (by COSMIC frequency).

Table S14 **Top focal regions of amplification and deletion among metastatic grade III ER+HER2- breast cancer cases from MSKCC cohort.**

| **Cytoband^a^** | **q value** | **Peak region (Mb) ^a^** | **Number of genes ^a,b^** | **Known proto-oncogene/ tumor suppressor gene in region ^a,b^** |
| --- | --- | --- | --- | --- |
| Amplification | | | | |
| 4p16.3 | **0.047074** | 0.00-5.84 | 86 | **FGFR3** |
| 8q24.13 | **0.047074** | 114.50-125.76 | 66 | **MYC** |
| 19p13.2 | 0.10821 | 5.03-7.45 | 71 | VAV1 |
| 20q13.2 | 0.10821 | 49.55-54.76 | 46 | GNAS |
| 14q21.1 | 0.1199 | 35.27-37.79 | 15 | FOXA1 |
| 12p13.1 | 0.15748 | 11.69-12.28 | 11 | CDKN1B |
| Deletion | | | | |
| 9p23 | **0.000181** | 5.31-15.43 | 27 | **PTPRD** |
| 11q23.2 | **0.001926** | 97.45-128.75 | 305 | **CBL** |
| 1p36.22 | **0.005287** | 9.33-14.59 | 77 | **MTOR** |
| 6q25.3 | **0.005959** | 151.03-163.19 | 85 | **QKI** |
| 19p13.3 | **0.009594** | 0.00-14.63 | 496 | **KEAP1** |
| 4q34.3 | **0.014627** | 149.61-182.30 | 139 | **FAT1** |
| 7q36.3 | **0.025721** | 145.11-151.77 | 26 | **MNX1** |
| 16q24.1 | **0.025721** | 74.44-85.43 | 98 | **CBFA2T3** |
| 14q13.3 | 0.05847 | 34.13-36.30 | 14 | FOXA1 |
| 2p23.2 | 0.061798 | 25.73-34.89 | 86 | ALK |
| 11p15.5 | 0.10077 | 0.00-6.92 | 224 | NUP98 |
| 13q13.1 | 0.13083 | 30.87-31.46 | 4 | BRCA2 |
| 12q24.33 | 0.23853 | 97.83-127.65 | 300 | TBX3 |

^a^Based on hg19 human genome assembly.

^b^RefSeq genes only.

^c^Known tumor suppressor genes and proto-oncogenes defined as found in COSMIC; if there is more than one known proto-oncogene in the region, only one is listed (priority for listing is, in order: known breast mutation; other known mutation (by COSMIC frequency).

Table S15. **Gene level CNA events from TCGA and METABRIC cohort.**

| Gene | Chromosome band | | Grade I/II ER+HER- | Grade III ER+HER- | TNBC | P-value^a^ | Grade I/II ER+HER- | Grade III ER+HER- | TNBC | P-value^a^ |
| --- | --- | --- | --- | --- | --- | --- | --- | --- | --- | --- |
|  |  |  | TCGA | | | | METABRIC | | | |
| CCND1 | 11q13.3 | Del^b^ | 23 (7.8) | 11 (12.6) | 25(20.8) | <0.001 | 21 (2.8) | 14 (3.5) | 17(6.8) | <0.001 |
|  |  | Gain | 47 (16.0) | 16 (18.2) | 36(30.0) |  | 48 (6.4) | 52(12.9) | 38(15.3) |  |
|  |  | Amp | 53 (18.1) | 20 (22.7) | 1 (0.8) |  | 99(13.3) | 108(26.8) | 15(6.0) |  |
| MYC | 8q24.21 | Del | 6 (2.0) | 2 (2.3) | 3 (2.5) | <0.001 | 9 (1.2) | 4 (1.0) | 5(2.0) | <0.001 |
|  |  | Gain | 118 (40.3) | 27 (30.7) | 67(55.8) |  | 133(17.8) | 97(24.1) | 69(27.7) |  |
|  |  | Amp | 21 (7.2) | 41 (46.6) | 35(29.2) |  | 93(12.4) | 113(28.0) | 97(39.0) |  |
| MDM2 | 12q15 | Del | 4 (1.4) | 7 (8.0) | 43(35.8) | <0.001 | 31 (4.1) | 19 (4.7) | 74(29.7) | <0.001 |
|  |  | Gain | 56 (19.1) | 20 (22.7) | 18(15.0) |  | 41 (5.5) | 52(12.9) | 11(4.4) |  |
|  |  | Amp | 8 (2.7) | 8 (9.1) | 1 (0.8) |  | 14 (1.9) | 23 (5.7) | 4(1.6) |  |
| CCNE1 | 19q12 | Del | 17 (5.8) | 16 (18.4) | 14(11.7) | <0.001 | 17 (2.3) | 24 (6.0) | 21(8.4) | 0.12 |
|  |  | Gain | 41 (14.0) | 16 (18.2) | 42(35.0) |  | 33 (4.4) | 30 (7.4) | 42(16.9) |  |
|  |  | Amp | 2 (0.7) | 5 (5.7) | 13(10.8) |  | 2 (0.3) | 7 (1.7) | 23(9.2) |  |

CNA: copy number alteration; ER: estrogen receptor; HER2: human epidermal growth factor receptor 2; METABRIC: Molecular Taxonomy of Breast Cancer International Consortium; PR: progesterone receptor; TCGA: the Cancer Genome Atlas; TNBC: triple negative breast cancer.

^a^ Pearson’s chi-square test or fisher’s exact test for grade III ER+HER- vs. grade I/II ER+HER- cases.

^b^ Del = homozygous deletion/hemizygous deletion.

Table S16**. Gene level CNA events from MSKCC and METABRIC cohort.**

| Gene | Chromosome band | | Grade I/II ER+HER- | Grade III ER+HER- | TNBC | P-value^a^ | Grade I/II ER+HER- | Grade III ER+HER- | TNBC | P-value^a^ |
| --- | --- | --- | --- | --- | --- | --- | --- | --- | --- | --- |
|  |  |  | MSKCC | | | | METABRIC | | | |
| FGFR3 | 4p16.3 | Del^b^ | 0 (0) | 0 (0) | 0 (0) | 0.55 | 51 (6.8) | 65(16.1) | 116(46.6) | <0.001 |
|  |  | Amp | 0 (0) | 4(1.5) | 0 (0) |  | 2 (0.3) | 5 (1.2) | 1(0.4) |  |
| MYC | 8q24.13 | Del | 0 (0) | 0 (0) | 0 (0) | <0.001 | 9 (1.2) | 4 (1.0) | 5(2.0) | <0.001 |
|  |  | Amp | 8 (3.1) | 11(42.3) | 7(10.9) |  | 93(12.4) | 113(28.0) | 97(39.0) |  |

CNA: copy number alteration; ER: estrogen receptor; HER2: human epidermal growth factor receptor 2; METABRIC: Molecular Taxonomy of Breast Cancer International Consortium; PR: progesterone receptor; MSKCC, Memorial Sloan Kettering Cancer Center.

^a^ Pearson’s chi-square test or fisher’s exact test for grade III ER+HER- vs. grade I/II ER+HER- cases.

^b^ Del = homozygous deletion/hemizygous deletion.

Table S17**. Univariate and multivariate analysis of amplification events by Cox proportional hazards models in grade III ER+HER2- tumors from TCGA and METABRIC cohorts.**

|  | Univariate competing risk model | | Univariate Cox proportional hazards regression | | Multivariate competing risk model | | Multivariate Cox proportional hazards regression | |
| --- | --- | --- | --- | --- | --- | --- | --- | --- |
|  | BCSS | | OS | | BCSS | | OS | |
|  | Hazard Ratio  (95% CI) | P value | Hazard Ratio  (95% CI) | P value | Hazard Ratio  (95% CI) | P value | Hazard Ratio  (95% CI) | P value |
| **CCND1** |  |  |  |  |  |  |  |  |
| No amp | 1.00(reference) |  | 1.00(reference) |  | / | / | / | / |
| Amp | 1.25(0.88, 1.8) | 0.22 | 1.12(0.85, 1.47) | 0.44 | / | / | / | / |
| **MYC** |  |  |  |  |  |  |  |  |
| No amp | 1.00(reference) |  | 1.00(reference) |  | 1.00(reference) |  | 1.00(reference) |  |
| Amp | 1.27(0.90,1.78) | 0.18 | **1.39(1.06, 1.81)** | **0.02** | 1.20(0.82,1.74) | 0.35 | 1.26(0.95,1.68) | 0.11 |
| **MDM2** |  |  |  |  |  |  |  |  |
| No amp | 1.00(reference) |  | 1.00(reference) |  | 1.00(reference) |  | 1.00(reference) |  |
| Amp | **1.69(1.15,2.48)** | **0.008** | 1.31(0.95, 1.81) | 0.10 | **1.72(1.17,2.53)** | **0.006** | **1.40(1.01,1.96)** | **0.045** |
| **CCNE1** |  |  |  |  |  |  |  |  |
| No amp | 1.00(reference) |  | 1.00(reference) |  |  |  |  |  |
| Amp | 1.24(0.76,2.01) | 0.38 | 1.16(0.77,1.75) | 0.47 |  |  |  |  |

Amp: amplification; BCSS: breast cancer-specific survival; ER: estrogen receptor; HER2: human epidermal growth factor receptor 2; HR: hazard ratio; METABRIC: Molecular Taxonomy of Breast Cancer International Consortium; OS: overall survival.

^a^Adjusted for age, stage, radiotherapy, endocrine therapy, chemotherapy and surgery.

Table S18. **Enriched pathways in grade III ER+HER2- tumors with MYC amplification in C2 sets (curated sets) by GSEA (NOM P<0.01, ES>0.6).**

| **Name** | **Size** | **NES** | **FDR** |
| --- | --- | --- | --- |
| NIKOLSKY_BREAST_CANCER_8Q23_Q24_AMPLICON | 64 | 2.59 | 0.00 |
| NIKOLSKY_BREAST_CANCER_8Q12_Q22_AMPLICON | 53 | 2.22 | 0.00 |
| CAIRO_HEPATOBLASTOMA_CLASSES_UP | 220 | 2.14 | 0.01 |
| PRAMOONJAGO_SOX4_TARGETS_DN | 27 | 2.08 | 0.02 |
| ELVIDGE_HIF1A_AND_HIF2A_TARGETS_UP | 18 | 2.06 | 0.02 |
| **KEGG_CELL_CYCLE** | 54 | 2.04 | 0.02 |
| RHODES_CANCER_META_SIGNATURE | 36 | 2.04 | 0.02 |
| SCHLOSSER_MYC_TARGETS_AND_SERUM_RESPONSE_UP | 19 | 2.02 | 0.03 |
| SHEDDEN_LUNG_CANCER_POOR_SURVIVAL_A6 | 193 | 1.97 | 0.04 |
| CHEMNITZ_RESPONSE_TO_PROSTAGLANDIN_E2_UP | 60 | 1.97 | 0.04 |
| SCHUHMACHER_MYC_TARGETS_UP | 35 | 1.93 | 0.07 |
| **VANTVEER_BREAST_CANCER_METASTASIS_DN** | 80 | 1.92 | 0.07 |
| MORI_PRE_BI_LYMPHOCYTE_UP | 49 | 1.90 | 0.07 |
| SHIPP_DLBCL_VS_FOLLICULAR_LYMPHOMA_UP | 34 | 1.90 | 0.07 |
| PEART_HDAC_PROLIFERATION_CLUSTER_DN | 30 | 1.84 | 0.10 |
| CHIN_BREAST_CANCER_COPY_NUMBER_UP | 19 | 1.84 | 0.10 |
| YOKOE_CANCER_TESTIS_ANTIGENS | 15 | 1.84 | 0.10 |
| SOTIRIOU_BREAST_CANCER_GRADE_1_VS_3_UP | 93 | 1.82 | 0.10 |
| HU_GENOTOXIC_DAMAGE_4HR | 17 | 1.76 | 0.12 |
| **SCIAN_CELL_CYCLE_TARGETS_OF_TP53_AND_TP73_DN** | 16 | 1.72 | 0.13 |
| HU_GENOTOXIC_DAMAGE_24HR | 18 | 1.70 | 0.14 |
| BERENJENO_ROCK_SIGNALING_NOT_VIA_RHOA_UP | 18 | 1.69 | 0.14 |
| YU_BAP1_TARGETS | 16 | 1.68 | 0.14 |
| KEGG_BLADDER_CANCER | 16 | 1.66 | 0.16 |

Abbreviations: NES, normalized enrichment score; FDR: false discovery rate.

Table S19**. Enriched pathways in III ER+HER2- tumors with MDM2 amplification in C2 sets (curated sets) by GSEA (NOM P<0.01, ES>0.7).**

| **Name** | **Size** | **NES** | **FDR** |
| --- | --- | --- | --- |
| REACTOME_UB_SPECIFIC_PROCESSING_PROTEASES | 54 | 2.15 | 0.018 |
| REACTOME_SWITCHING_OF_ORIGINS_TO_A_POST_REPLICATIVE_STATE | 36 | 2.05 | 0.028 |
| REACTOME_SCF_SKP2_MEDIATED_DEGRADATION_OF_P27_P21 | 27 | 2.03 | 0.022 |
| **REACTOME_CELL_CYCLE_CHECKPOINTS** | 90 | 2.03 | 0.021 |
| REACTOME_G1_S_DNA_DAMAGE_CHECKPOINTS | 27 | 2.03 | 0.022 |
| REACTOME_ORC1_REMOVAL_FROM_CHROMATIN | 31 | 2.02 | 0.023 |
| REACTOME_APC_C:CDH1_MEDIATED_DEGRADATION_OF_CDC20_AND_OTHER_APC_C:CDH1_TARGETED_PROTEINS_IN_LATE_MITOSIS_EARLY_G1 | 29 | 2.01 | 0.022 |
| REACTOME_FBXL7_DOWN_REGULATES_AURKA_DURING_MITOTIC_ENTRY_AND_IN_EARLY_MITOSIS | 24 | 2.01 | 0.021 |
| REACTOME_UCH_PROTEINASES | 38 | 2.01 | 0.021 |
| REACTOME_CDK_MEDIATED_PHOSPHORYLATION_AND_REMOVAL_OF_CDC6 | 27 | 1.99 | 0.023 |
| REACTOME_REGULATION_OF_PTEN_STABILITY_AND_ACTIVITY | 23 | 1.99 | 0.023 |
| REACTOME_DEGRADATION_OF_DVL | 23 | 1.98 | 0.024 |
| REACTOME_NEGATIVE_REGULATION_OF_NOTCH4_SIGNALING | 25 | 1.98 | 0.023 |
| REACTOME_DNA_REPLICATION | 46 | 1.98 | 0.023 |
| REACTOME_REGULATION_OF_MITOTIC_CELL_CYCLE | 38 | 1.97 | 0.023 |
| REACTOME_CELLULAR_RESPONSE_TO_HYPOXIA | 28 | 1.97 | 0.024 |
| REACTOME_REGULATION_OF_RUNX3_EXPRESSION_AND_ACTIVITY | 23 | 1.96 | 0.024 |
| REACTOME_DEGRADATION_OF_AXIN | 22 | 1.95 | 0.025 |
| REACTOME_ASSEMBLY_OF_THE_PRE_REPLICATIVE_COMPLEX | 30 | 1.95 | 0.025 |
| REACTOME_ACTIVATION_OF_APC_C_AND_APC_C:CDC20_MEDIATED_DEGRADATION_OF_MITOTIC_PROTEINS | 32 | 1.94 | 0.026 |
| REACTOME_VIF_MEDIATED_DEGRADATION_OF_APOBEC3G | 24 | 1.93 | 0.028 |
| REACTOME_DNA_REPLICATION_PRE_INITIATION | 33 | 1.93 | 0.029 |
| REACTOME_METABOLISM_OF_POLYAMINES | 26 | 1.92 | 0.030 |
| **REACTOME_STABILIZATION_OF_P53** | 22 | 1.92 | 0.030 |
| REACTOME_REGULATION_OF_APOPTOSIS | 21 | 1.91 | 0.033 |
| REACTOME_RUNX1_REGULATES_TRANSCRIPTION_OF_GENES_INVOLVED_IN_DIFFERENTIATION_OF_HSCS | 49 | 1.90 | 0.033 |
| REACTOME_CROSS_PRESENTATION_OF_SOLUBLE_EXOGENOUS_ANTIGENS_ENDOSOMES | 19 | 1.90 | 0.032 |
| REACTOME_DEFECTIVE_CFTR_CAUSES_CYSTIC_FIBROSIS | 22 | 1.90 | 0.032 |
| KEGG_PROTEASOME | 17 | 1.89 | 0.037 |
| REACTOME_SUMOYLATION_OF_CHROMATIN_ORGANIZATION_PROTEINS | 15 | 1.85 | 0.046 |
| PELLICCIOTTA_HDAC_IN_ANTIGEN_PRESENTATION_DN | 25 | 1.82 | 0.059 |
| REACTOME_MITOTIC_PROPHASE | 39 | 1.77 | 0.092 |
| HU_GENOTOXIC_DAMAGE_4HR | 17 | 1.76 | 0.094 |
| REACTOME_CHROMOSOME_MAINTENANCE | 32 | 1.74 | 0.104 |
| REACTOME_FORMATION_OF_THE_BETA_CATENIN:TCF_TRANSACTIVATING_COMPLEX | 33 | 1.73 | 0.105 |

Abbreviations: NES, normalized enrichment score; FDR: false discovery rate.

Table S20**. Differential gene expression (DGE) in non-luminal like compared with luminal like grade III ER+HER2- cases.**

| Gene | logFC | FDR | Correlation coefficient | P value of correlation test |
| --- | --- | --- | --- | --- |
| Lowly expressed in non-luminal like (logFC>4) | | | | |
| FABP7 | -5.69093759 | 1.56E-12 | 0.755416 | 7.42E-15 |
| SLC6A14 | -5.588645055 | 5.46E-11 | 0.757716 | 4.72E-06 |
| A2ML1 | -5.457678743 | 4.47E-08 | 0.591047 | 2.96E-08 |
| KRT16 | -5.349362892 | 7.65E-07 | 0.56055 | 2.06E-07 |
| KRT6A | -5.135812109 | 1.28E-05 | 0.514818 | 2.70E-06 |
| KRT6B | -5.084207198 | 1.01E-05 | 0.574838 | 8.50E-08 |
| VGLL1 | -5.08326044 | 2.87E-09 | 0.766803 | 0.001375 |
| SERPINB5 | -4.911008458 | 8.56E-07 | 0.791312 | 4.85E-17 |
| PPP1R14C | -4.803952238 | 2.04E-10 | 0.614403 | 1.39E-07 |
| PI3 | -4.792158923 | 9.79E-09 | 0.726094 | 0.000432 |
| KRT5 | -4.676716671 | 0.00015 | 0.731272 | 1.38E-13 |
| TRIM29 | -4.67276682 | 5.65E-07 | 0.666656 | 8.94E-11 |
| MIA | -4.594255728 | 2.64E-07 | 0.792746 | 6.51E-10 |
| SFRP1 | -4.531467969 | 1.36E-06 | 0.742836 | 3.54E-14 |
| BBOX1 | -4.468303891 | 3.87E-09 | 0.661725 | 2.81E-08 |
| DSG1 | -4.452441437 | 5.83E-08 | 0.664695 | 0.025662 |
| DSG3 | -4.327923901 | 0.00018 | 0.560435 | 3.43E-05 |
| S100A7 | -4.240221001 | 0.0111343 | 0.732405 | 1.21E-13 |
| SBSN | -4.181097413 | 1.63E-06 | 0.58272 | 0.000192 |
| CRABP1 | -4.039190096 | 0.0015 | 0.721393 | 4.17E-13 |
| Lowly expressed in non-luminal like (logFC<-3) | | | | |
| AGR3 | -6.202407725 | 5.26E-11 | 0.812258894 | 1.60E-18 |
| TFF1 | -6.052270342 | 2.57E-07 | 0.705940345 | 6.17E-12 |
| TFF3 | -5.725008736 | 2.70E-09 | 0.741285808 | 4.27E-14 |
| FOXA1 | -5.109074769 | 2.64E-17 | 0.723343112 | 3.36E-13 |
| ESR1 | -5.093943998 | 3.77E-16 | 0.760342737 | 3.92E-15 |
| AGR2 | -5.000188845 | 4.11E-10 | 0.777033901 | 4.01E-16 |
| PGR | -4.414297847 | 2.86E-05 | 0.734360524 | 9.65E-14 |
| GFRA1 | -4.316868638 | 1.16E-06 | 0.751466332 | 1.22E-14 |
| SCUBE2 | -4.312956648 | 5.83E-08 | 0.636614394 | 1.08E-09 |
| VSTM2A | -4.056317258 | 0.005 | 0.70537519 | 0.015 |
| DNALI1 | -4.002024085 | 6.09E-10 | 0.693443377 | 6.16E-09 |
| DHRS2 | -3.93581658 | 0.004 | 0.64303889 | 6.50E-10 |
| AKR7A3 | -3.85908416 | 2.56E-06 | 0.770115876 | 1.06E-15 |
| ANKRD30A | -3.827371943 | 0.007 | 0.508228606 | 0.0001 |
| KLHDC7A | -3.826495841 | 0.0006 | 0.68063467 | 3.01E-09 |
| SPDEF | -3.767379733 | 8.25E-11 | 0.602460757 | 1.36E-08 |
| NAT1 | -3.628679372 | 7.06E-06 | 0.78795367 | 8.09E-17 |
| PRR15 | -3.604637379 | 5.69E-09 | 0.680193324 | 1.77E-10 |
| THSD4 | -3.601332153 | 9.23E-11 | 0.656452861 | 2.15E-10 |
| AR | -3.518843389 | 1.30E-07 | 0.619896929 | 3.88E-09 |
| GP2 | -3.504418551 | 0.001 | 0.684744538 | 1.73E-11 |
| RET | -3.427874507 | 2.99E-05 | 0.641392807 | 0.0031 |
| CLSTN2 | -3.421660007 | 9.11E-07 | 0.744016221 | 3.07E-14 |
| ANXA9 | -3.402477609 | 1.43E-11 | 0.560021052 | 2.13E-07 |
| MLPH | -3.266500353 | 3.09E-13 | 0.660212914 | 1.56E-10 |
| GREB1 | -3.240016905 | 5.04E-07 | 0.70099546 | 1.99E-11 |
| GATA3 | -3.190995192 | 6.06E-13 | 0.859984639 | 1.00E-22 |

Abbreviations: logFC: log2 (fold change); FDR: false discovery rate.

Table S21**. Differential pathways in non-luminal grade III ER+HER2- tumors in C2 sets (curated sets) by GSVA (FDR<0.05).**

| Name | FDR | LogFC |
| --- | --- | --- |
| **Upregulated pathways in non-luminal like cases (logFC>0.3)** | | |
| BIOCARTA_THELPER_PATHWAY | 0.0003 | 0.31 |
| VANTVEER_BREAST_CANCER_ESR1_DN | 1.11E-17 | 0.31 |
| REACTOME_TNF_RECEPTOR_SUPERFAMILY_TNFSF_MEMBERS_MEDIATING_NON_CANONICAL_NF_KB_PATHWAY | 3.47E-10 | 0.31 |
| BIOCARTA_TCYTOTOXIC_PATHWAY | 0.0003 | 0.31 |
| BIOCARTA_TCRA_PATHWAY | 0.0002 | 0.31 |
| BIOCARTA_CTL_PATHWAY | 3.98E-05 | 0.32 |
| YANG_BREAST_CANCER_ESR1_DN | 5.26E-14 | 0.33 |
| SMID_BREAST_CANCER_LUMINAL_B_DN | 5.87E-15 | 0.33 |
| SOUCEK_MYC_TARGETS | 1.32E-10 | 0.34 |
| FINETTI_BREAST_CANCER_KINOME_GREEN | 3.93E-07 | 0.35 |
| SMID_BREAST_CANCER_RELAPSE_IN_BRAIN_UP | 3.44E-24 | 0.36 |
| DOANE_BREAST_CANCER_ESR1_DN | 8.02E-22 | 0.39 |
| **Downregulated pathways in non-luminal like cases (logFC< -0.3)** | | |
| FARMER_BREAST_CANCER_CLUSTER_6 | 9.32E-24 | 0.49 |
| YANG_BREAST_CANCER_ESR1_UP | 2.34E-20 | 0.42 |
| SMID_BREAST_CANCER_RELAPSE_IN_LIVER_DN | 3.68E-15 | 0.39 |
| VANTVEER_BREAST_CANCER_ESR1_UP | 1.12E-25 | 0.40 |
| YANG_BREAST_CANCER_ESR1_BULK_UP | 3.74E-25 | 0.39 |
| DOANE_BREAST_CANCER_ESR1_UP | 1.21E-21 | 0.34 |
| LIEN_BREAST_CARCINOMA_METAPLASTIC_VS_DUCTAL_DN | 4.10E-22 | 0.33 |
| SMID_BREAST_CANCER_LUMINAL_B_UP | 5.06E-22 | 0.33 |
| YANG_BREAST_CANCER_ESR1_LASER_UP | 7.29E-15 | 0.33 |
| SMID_BREAST_CANCER_RELAPSE_IN_BRAIN_DN | 1.93E-19 | 0.32 |

Abbreviations: LogFC: log2 (fold change); FDR: false discovery rate.

Table S22**. Enriched pathways in non-luminal grade III ER+HER2- tumors in C2 sets (curated sets) by GSEA (NOM P<0.05).**

| Name | Size | NES | FDR |
| --- | --- | --- | --- |
| KEGG_EPITHELIAL_CELL_SIGNALING_IN_HELICOBACTER_PYLORI_INFECTION | 67 | 1.80 | 0.78 |
| KEGG_GLYCOSPHINGOLIPID_BIOSYNTHESIS_LACTO_AND_NEOLACTO_SERIES | 25 | 1.74 | 0.67 |
| KEGG_DORSO_VENTRAL_AXIS_FORMATION | 24 | 1.69 | 0.66 |
| KEGG_LYSINE_DEGRADATION | 39 | 1.69 | 0.50 |
| KEGG_NON_SMALL_CELL_LUNG_CANCER | 54 | 1.69 | 0.40 |
| KEGG_NOD_LIKE_RECEPTOR_SIGNALING_PATHWAY | 60 | 1.68 | 0.36 |
| KEGG_ERBB_SIGNALING_PATHWAY | 86 | 1.59 | 0.58 |
| KEGG_CHEMOKINE_SIGNALING_PATHWAY | 184 | 1.58 | 0.56 |
| KEGG_P53_SIGNALING_PATHWAY | 65 | 1.56 | 0.51 |
| KEGG_VIBRIO_CHOLERAE_INFECTION | 53 | 1.55 | 0.47 |
| KEGG_SMALL_CELL_LUNG_CANCER | 84 | 1.53 | 0.43 |
| KEGG_PRION_DISEASES | 35 | 1.52 | 0.43 |
| KEGG_CYTOKINE_CYTOKINE_RECEPTOR_INTERACTION | 257 | 1.52 | 0.41 |

Table S23**. Clinicopathological characteristics of Luminal-like and Non-luminal-like tumors within grade III ER+ HER2- breast cancer patients of Chinese IHC-based cohort.**

|  | **Luminal-like** | **Non-luminal-like** | P-value^a^ |
| --- | --- | --- | --- |
|  | **N=63** | **N=18** |  |
| **Age (mean±SD)** | 47.75 (10.71) | 46.22 (11.43) | 0.6 |
| **T stage** |  |  |  |
| T1 | 37 (58.7%) | 5 (27.8%) | 0.02 |
| T2 | 24 (38.1%) | 10 (55.6%) |  |
| T3-4 | 2 (3.2%) | 3 (16.7%) |  |
| **N stage** |  |  |  |
| N0 | 33 (52.4%) | 5 (27.8) | 0.04 |
| N1-3 | 30 (47.6%) | 13 (72.2) |  |
| **Surgery** |  |  |  |
| BCS | 9 (14.3%) | 4 (22.2%) | 0.68 |
| Mastectomy | 40 (63.5%) | 11 (61.1%) |  |
| Others and Unknown | 14 (22.2%) | 3 (16.7%) |  |
| **Chemotherapy** |  |  |  |
| Yes | 63 (100%) | 18 (100%) | / |
| **Endocrine therapy** |  |  |  |
| Yes | 63 (100%) | 18 (100%) | / |

Abbreviations: SD, standard deviation; BCS, breast conserving surgery.

^a^Pearson’s chi-square, Fisher’s exact and T test was performed between the Luminal-like and Non-luminal-like groups if needed.
